# Glutaminase‐1 Mediated Glutaminolysis to Glutathione Synthesis Maintains Redox Homeostasis and Modulates Ferroptosis Sensitivity in Cancer Cells

**DOI:** 10.1111/cpr.70036

**Published:** 2025-04-21

**Authors:** Changsen Bai, Jialei Hua, Donghua Meng, Yue Xu, Benfu Zhong, Miao Liu, Zhaosong Wang, Wei Zhou, Liming Liu, Hailong Wang, Yang Liu, Lifang Li, Xiuju Chen, Yueguo Li

**Affiliations:** ^1^ Department of Clinical Laboratory National Clinical Research Center for Cancer, Key Laboratory of Cancer Prevention and Therapy, Tianjin's Clinical Research Center for Cancer, Tianjin Medical University Cancer Institute and Hospital Tianjin China; ^2^ Academy of Medical Engineering and Translational Medicine, Tianjin University Tianjin China; ^3^ Department of Radiology National Clinical Research Center for Cancer, Key Laboratory of Cancer Prevention and Therapy, Tianjin's Clinical Research Center for Cancer, Tianjin Medical University Cancer Institute and Hospital Tianjin China; ^4^ Department of Cancer Cell Biology National Clinical Research Center for Cancer, Key Laboratory of Cancer Prevention and Therapy, Tianjin's Clinical Research Center for Cancer, Tianjin Medical University Cancer Institute and Hospital Tianjin China; ^5^ Department of Pediatric Oncology National Clinical Research Center for Cancer, Key Laboratory of Cancer Prevention and Therapy, Tianjin's Clinical Research Center for Cancer, Tianjin Medical University Cancer Institute and Hospital Tianjin China; ^6^ Department of Radiotherapy National Clinical Research Center for Cancer, Key Laboratory of Cancer Prevention and Therapy, Tianjin's Clinical Research Center for Cancer, Tianjin Medical University Cancer Institute and Hospital Tianjin China; ^7^ Laboratory Animal Center, National Clinical Research Center for Cancer, Key Laboratory of Cancer Prevention and Therapy, Tianjin's Clinical Research Center for Cancer, Tianjin Medical University Cancer Institute and Hospital Tianjin China; ^8^ Department of Public Laboratory National Clinical Research Center for Cancer, Key Laboratory of Cancer Prevention and Therapy, Tianjin's Clinical Research Center for Cancer, Tianjin Medical University Cancer Institute and Hospital Tianjin China; ^9^ Department of Hepatobiliary Cancer Liver Cancer Center, Tianjin Medical University Cancer Institute and Hospital, National Clinical Research Center for Cancer, Key Laboratory of Cancer Prevention and Therapy, Tianjin's Clinical Research Center for Cancer Tianjin China; ^10^ Department of Hepatobiliary and Pancreatic Oncology, Tianjin Cancer Hospital Airport Hospital Tianjin China; ^11^ Department of Neurology Tianjin Nankai Hospital, Tianjin Medical University Tianjin China

**Keywords:** cancer cell, ferroptosis, GLS1, glutamate, GPX4, GSH

## Abstract

Glutaminase‐1 (GLS1) has garnered considerable interest as a metabolic target in cancer due to its heightened involvement and activity. However, the precise fate of glutaminolysis catalysed by GLS1 in cancer cells remains elusive. We found that GLS1 knockout led to significant suppression of cancer cell proliferation, which can be reversed or partially restored by supplementation of glutamate or non‐essential amino acids that can be converted into glutamate. The addition of spliceosomal KGA or GAC ameliorates cancer cell growth in vitro and in vivo, providing both simultaneously completely reverse the effect. The primary metabolic fate of glutamate produced through glutaminolysis in cancer cells is mainly used to produce glutathione (GSH) for redox homeostasis, not entering the tricarboxylic acid cycle or synthesising nucleotides. GSH monoethyl ester (GSH‐MEE) effectively rescues the inhibition of cancer cell proliferation caused by GLS1 knockout. Deletion of GLS1 results in an elevation of reactive oxygen species (ROS) and malondialdehyde (MDA), a reduction of NADPH/NADP^+^ ratio, and an augmented susceptibility of cells to ferroptosis. Glutathione Peroxidase 4 (GPX4) and GPX1 exhibit complementary roles in redox regulation, with GLS1 knockout promoting GPX4 degradation. Pharmacological inhibition of GLS1 synergises with GPX4 inhibitor to suppress tumour growth. Dual targeting of GPX4 and GPX1 presents a potent anti‐cancer strategy. This metabolic mechanism facilitates a deeper comprehension of the abnormal glutamine metabolism in cancer cells, establishing a theoretical basis for the potential clinical utilisation of GLS1 inhibitors and presenting novel perspectives for advancing combinatorial therapeutic approaches.

## Graphical abstract

GLS1‐mediated glutaminolysis supports GSH synthesis in cancer cells. GLS1 KO increases ROS, downregulates GPX4, and upregulates GPX1, making cells more sensitive to ferroptosis. Combining GLS1 or GPX1 inhibitors with a GPX4 inhibitor synergistically suppresses cancer growth. GLS1 KO also reduces AMPK activity, might enhance ferroptosis sensitivity.

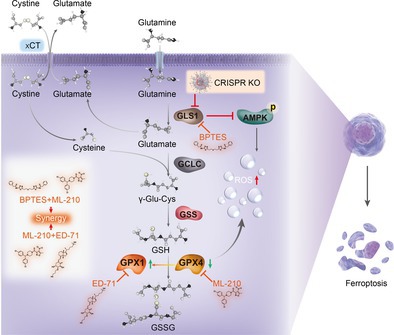

## 1 Introduction

Metabolic abnormalities are hallmarks of cancer [1], with the metabolic transformation of cancer cells being potential therapeutic targets. Otto Warburg's research on aerobic glycolysis highlighted glucose as central to cancer metabolism studies [2]. Glutamine, another key nutrient, supports survival and biosynthesis in cancer cells by providing carbon and nitrogen for macromolecule production and powering the tricarboxylic acid (TCA) cycle [3, 4]. Controlled oxidation of glutamine harnesses nicotinamide adenine dinucleotide (reduced form‐NADH) and flavin adenine dinucleotide (reduced form‐FADH2)'s reducing power, generating adenosine triphosphate (ATP) [5]. Glutamine nitrogen is essential for cancer cell proliferation under normoxia [6]. Ammonia from glutaminolysis recycles to produce glutamate, proline and aspartate for biomass, especially in breast cancer cells [7]. Under hypoxia or with impaired mitochondrial function, cells increase glutamine consumption [8]. Reductive carboxylation of glutamine supplies carbon for fatty acid biosynthesis [9, 10], with excess carbon and nitrogen expelled as dihydroorotate [6]. The role of glutaminolysis, catalysed by glutaminase (GLS1), in cancer cells remains incompletely elucidated.

GLS is an attractive metabolic target in cancer due to its enhanced role and activity [11]. Two types of GLS are expressed in mammalian cells: GLS1 (kidney type) and GLS2 (liver type). GLS1 is upregulated in cancer, whilst GLS2 is downregulated. Alternative splicing of GLS1 mRNA produces two isoforms, GAC and KGA, with GAC having high basal activity in cancer [3]. CB‐839, a specific GLS1 inhibitor, is in clinical trials for various cancers, either alone or in combination [4]. However, GLS1's clinical effectiveness is limited by the lack of reliable predictive and pharmacodynamic markers, restricting its usefulness. Understanding GLS1‐mediated glutamine catabolism in cancer is essential for improving its therapeutic implementation and developing combination treatments.

Ferroptosis, unlike apoptosis, necroptosis and autophagy, is an iron‐dependent programmed cell death triggered by chronic and acute metabolic stress [12, 13]. Depletion of glutathione (GSH), reduced Glutathione Peroxidase 4 (GPX4) activity, diminished antioxidant capacity and elevated reactive oxygen species (ROS) lead to lipid peroxidation and metabolic impairment, inducing ferroptosis [14, 15]. The cystine/GSH/GPX4 system is a primary defence against ferroptosis [15, 16]. Cysteine, the primary intracellular antioxidant, is essential for GSH production, a tripeptide of cysteine, glutamate and glycine [17, 18]. Cancer cells obtain cystine from the environment via the cystine/glutamate antiporter SLC7A11 (xCT), which exchanges extracellular cystine for intracellular glutamate. Cystine is then reduced to cysteine using nicotinamide adenine dinucleotide phosphate (reduced form‐NADPH) [19]. Impaired cystine uptake leads to glutamate buildup [20], but glutamate is also necessary for GSH biosynthesis. The role of GLS1 in providing glutamate for GSH or cystine exchange in cancer cells is unclear, as is the maintenance of redox balance across different cancer types.

In our study, GLS1 was knocked out in multiple cancer cell lines using CRISPR/Cas9 to characterise its role. Our results showed that GLS1 removal significantly impeded the growth of MCF‐7, HCT116 and LN229 cell lines. Glutamate addition rescued cell proliferation, and non‐essential amino acids partially rescued it. The adding back of KGA or GAC largely rescued proliferation, whilst their simultaneous supplementation ultimately rescued it. Metabolomics, transcriptomics, metabolic flux and rescue experiments revealed that GSH, but not phosphatidylglycerol (PG) or phosphatidylcholine (PC), rescued the proliferation inhibition caused by GLS1 knockout. GLS1 knockout impaired antioxidant capacity and increased susceptibility to ferroptosis in cancer cells. GPX4 and GPX1 exhibit complementary roles in redox regulation, with GLS1 knockout promoting GPX4 degradation. The GLS1 inhibitor synergizes with the GPX4 inhibitor to inhibit tumour growth, and dual suppression of GPX4 and GPX1 offers a potent anti‐cancer strategy.

## 2 Materials and Methods

### 2.1 Cell Culture and Reagents

MCF‐7, HCT116, LN229 and 293 T cells were cultured in DMEM containing 10% fetal bovine serum (BioInd, Israel) and 50 IU penicillin/streptomycin (Solarbio, China). Cell lines were acquired from ATCC, cultured at 37°C in a humidified environment with 5% CO2, and confirmed to be free of mycoplasma contamination. The abbreviations used in this study are listed in Table 1, and the reagents used are shown in Table 2.

**TABLE 1:** Abbreviations used in this study.

Abbreviation	Name
GLS1	Glutaminase‐1
KGA	Kidney glutaminase
GAC	Glutaminase C
GSH	Glutathione
GSH‐MEE	GSH monoethyl ester
ROS	Reactive oxygen species
MDA	Malondialdehyde
TCA	Tricarboxylic acid
NADH	Nicotinamide adenine dinucleotide, reduced form
FADH2	Flavin adenine dinucleotide, reduced form
ATP	Adenosine triphosphate
NADPH	Nicotinamide adenine dinucleotide phosphate, reduced form
GPX4	Glutathione peroxidase 4
GPX1	Glutathione peroxidase 1
PG	Phosphatidylglycerol
PC	Phosphatidylcholine
DCFH‐DA	Dilute dichlorofluorescein diacetate
GSSG	Glutathione disulphide
DMαKG	Dimethyl α‐Ketoglutarate
GSDMC	Gasdermin C
NEAAs	Non‐essential amino acids
AKB	2‐ketobutyric acid
GABA	γ‐aminobutyric acid
AntA	Antimycin A
CAD	Carbamoyl‐phosphate synthetase II, aspartate transcarbamylase, and dihydroorotase
MSEA	Metabolite sets enrichment analysis
LPCAT1	Lysophosphatidylcholine acyltransferase 1
U	Uridine
A	Adenosine
G	Guanosine
C	Cytidine
IMP	Inosine monophosphate
dCMP	Deoxycytidine‐monophosphate
NAC	*N*‐acetylcysteine
GCLC	Glutamate‐cysteine ligase catalytic subunit
ED‐71	Eldecalcitol
DMFS	Distant metastasis free survival
RFS	Recurrence free survival
IC50	Half‐maximal inhibitory concentration
H_2_O_2_	Hydrogen peroxide

**TABLE 2:** Reagents used in this study.

Name	CAS number	Source	Product number
Dimethyl Α‐ketoglutarate	5968‐43‐0	Sigma	349,631
*N*‐acetyl‐L‐cysteine	616‐91‐1	Sigma	A9165
Non‐essential amino acid mix	Mixture	Sigma	M7145
Lipid mixture	Mixture	Sigma	L0288
Adenosine	58‐61‐7	Sigma	A4036
Guanosine	118‐00‐3	Sigma	G264
Cytidine	71‐30‐7	Sigma	C4654
Uridine	58‐96‐8	Sigma	U3003
Glutathione	70‐18‐8	Sigma	PHR1359
Glutathione monoethyl ester	56‐89‐3	Sigma	353,905
2‐Ketobutyric acid	513‐74‐4	Sigma	K401
γ‐Aminobutyric acid	1956/12/2	Sigma	A2129
All amino acids	N/A	Sigma	N/A
Phosphatidylcholine (PC)	1957/11/4	Aladdin	L130331
Phosphatidylglycerol (PG)	1957/10/3	Aladdin	L130372
BPTES	314045‐39‐1	MCE	HY‐12683
ML‐210	1360705‐96‐9	MCE	HY‐100003
ED‐71	104121‐92‐8	MCE	HY‐A0020
CCK‐8	N/A	TargetMol	C0005

### 2.2 Generation of GLS1 Knock Down and Knock Out Cell Lines

shRNA cloned into the pLKO.1 vector was used to knock down GLS1, with sequences including shGLS1‐1: CCATAAGAATCTTGATGGATT, shGLS1‐2: CCTCACACATTGATGAGTTAT. A CRISPR‐Cas9 system was used to eliminate GLS1 from MCF‐7, HCT116, or LN229 cells using pCDH‐Cas9‐2A‐GFP‐BSD for Cas9 expression and BsmBI‐linearized pLentiGuide‐Puro vector for sgRNAs. The GeCKO v2 library was used for the target sequence of GLS1 [21]. The sgRNA targeting human GLS1 was sgGLS1‐5, 5'‐AACAGCAAATCTTCCAAGCT‐3′. Cells were transfected with pCDH‐Cas9‐2A‐GFP‐BSD and pLentiGuide‐Puro‐sgGLS1 plasmids using Lipofectamine 3000. Single cells were sorted into 96 wells with 200 μL DMEM supplemented with 10% FBS using a flow cytometer detecting green fluorescence. After 3 weeks, colonies were trypsinised, expanded, and validated for GLS1 knockout via Western blot.

### 2.3 Proliferation Assay

Cells (2 × 10^4^) were seeded in triplicate in 24‐well plates with 1 mL of DMEM and 10% dialyzed fetal bovine serum (pyruvate‐free). The initial cell count was determined by measuring 3 wells at the start of treatment. Cells were washed twice with PBS, treated with 1 mL of treatment media, and then fixed with 4% paraformaldehyde in PBS for 30 min at room temperature. Wells were stained with 1 μg/mL of Hoechst 33345, and cell numbers were determined using the High Content Analysis System (PE, Operetta CLS) with images taken by the Operetta CLS microscope. The counting technique was validated against a haemocytometer and the Cell Titre‐Glo Luminescent Cell Viability Assay Kit (Cat #7570; Promega).

### 2.4 Drug Matrix Design and CCK‐8 Assay

The cell proliferation under combination drug treatment was assessed using the CCK‐8 assay. Cells were seeded in 96‐well plates at a density of 3 × 10^3^ cells per well and allowed to adhere overnight. Different concentrations of drugs (single agent or dual‐drug combination) were then added, followed by incubation for 72 h. Afterward, CCK‐8 reagent was added and incubated for 2 h, and the absorbance at a wavelength of 450 nm was measured to evaluate cell viability. The synergy of drug combinations was quantified using SynergyFinder (https://synergyfinder.fimm.fi/), which calculates the Synergy Score based on the percentage of cell viability. The synergy was assessed using the formula: *E*_HSA_ = *E*_AB_ − max (*E*_A_, *E*_B_), where *E*_AB_ represents the observed effect of the drug combination (drugs A and B combined); *E*_A_ is the effect of drug A alone; *E*_B_ is the effect of drug B alone; max (*E*_A_, *E*_B_) is the larger effect observed with either drug A or drug B alone. A positive *E*_HSA_ score greater than 10 indicates a synergistic interaction between the drugs.

### 2.5 NADPH/NADP ^+^ Ratio Assay

The NADPH/NADP^+^ ratio was calculated using the NADPH/NADP‐Glo Assay (Cat#G9082, Promega). Cells (150,000) were placed in 12‐well plates, incubated with treatment medium, washed with PBS, and extracted with 400 μL ice‐cold lysis buffer. Samples (150 μL) were incubated at 60°C for 15 min to measure NADPH and treated with 0.4 N HCl for NADP^+^ measurement. After neutralisation, samples were mixed with NADP^+^/NADPH‐Glo Detection Reagent in a 384‐well solid black luminometer plate. Luminescence was measured using the Synergy H1 Hybrid Multi‐Mode reader (BioTek).

### 2.6 Colony Formation Assay

Cells (1000) were placed in 12‐well plates and incubated with 2 mL of culture medium for 2 weeks. Cells were then fixed with 4% paraformaldehyde in PBS and stained with 0.5% crystal violet for 5 min.

### 2.7 Western Blot

Cells were lysed in buffer (20 mM Tris–HCl, pH 7.5, 150 mM NaCl, 1 mM EDTA, 1% Triton X‐100, 2.5 mM sodium pyrophosphate, 1 mM β‐glycerophosphate, 1 mM sodium vanadate, 1 mg/mL leupeptin, 1 mM phenylmethylsulfonylfluoride). Proteins were resolved on 10% SDS‐PAGE gels and detected using the BLT GelView 6000 Plus image reader. Primary antibodies and secondary antibodies are shown in Table 3.

**TABLE 3:** Antibodies used in this study.

Name	Source	Identifier	Usage
β‐Actin	Proteintech (US)	60008‐1	1:5000
GAPDH	Proteintech (US)	60004‐1‐Ig	1:5000
GCLC	Proteintech (US)	12601‐1‐AP	1:1000
AMPK	Abcam (UK)	ab32047	1:1000
GPX1	Abgent (US)	AP9315D‐ev	1:1000
GSDME	Abcam (UK)	ab215191	1:1000
GLS1	CST (US)	49363	1:1000
GPX4	CST (US)	59735S	1:1000
p‐AMPK	CST (US)	2535	1:1000
PARP1	CST (US)	9542	1:1000
Caspase‐3	Santa Cruz (US)	sc‐56046	1:1000
LC3	Santa Cruz (US)	sc‐292354	1:1000
Goat anti‐rabbit IgG secondary antibody(HRP)	Abgent(US)	LP1001a	1:10000
Goat anti‐mouse IgG secondary antibody (HRP)	ZSGB‐BIO(CN)	ZB‐2305	1:10000

### 2.8 Gene Construction and Lentivirus Production

Lentiviral vectors pCDH‐puro‐CMV or pCDH‐Neo‐CMV were used for cDNA cloning. 293 T cells were transfected with expression plasmids, pCMV‐dR8.91 and pCMV‐VSV‐G, using PEI. The virus‐containing medium was filtered and stored at −80°C. Cancer cells were exposed to viruses with polybrene (10 μg/mL) and selected with puromycin or neomycin.

### 2.9 Sphere Formation Experiment

Cells (1000) were plated in a low‐adsorption culture plate (Corning, #3473) with DMEM supplemented with EGF, FGF and B27.

### 2.10 Animal Experiments

Animal protocols were approved by the Institutional Animal Care and Use Committee (IACUC) of Tianjin Medical University (approval number AE‐2021102). Nude mice (4–5 weeks old, 19–20 g) from Jiangsu GemPharmatech Biotechnology Co. were housed in an SPF environment. Mice were injected with 5 × 10^6^ cells in 100 μL PBS (*n* = 6 mice per group). Tumour growth was measured every 3 days with digital callipers, and after 6 weeks, mice were euthanized for further analysis.

### 2.11 Glutamate Detection Assay

Cells (10^6^) were seeded in 6‐well dishes and incubated for 8 h with fresh DMEM. Glutamate levels were assessed using the Abcam Glutamate Assay Kit (#ab83389) following the manufacturer's guidelines.

### 2.12 Detection of Intracellular ROS

Intracellular ROS was determined using a cell‐based ROS assay kit from Beyotime Biotechnology with 10 μM dilute dichlorofluorescein diacetate (DCFH‐DA). 5 × 10^5^ cells were cultured for 20 h in 6‐well plates. Cells were incubated with DCFH‐DA for 20 min at 37°C, washed with serum‐free medium, and fluorescence images were captured using an EVOS inverted fluorescence microscope.

### 2.13 Detection of Intracellular GSH and Glutathione Disulphide (GSSG)

10^6^ cells were seeded in 6‐well plates and incubated for 20 h to measure the levels of GSH and GSSG inside the cells (Beyotime Biotechnology). The cells were washed with PBS twice and trypsinized to collect cellular pellets. Cellular pellets (10 μL or 10 mg) were combined with 30 μL of 5% protein removal reagent M from the GSH and GSSG assay kit, followed by two quick freeze–thaw cycles using liquid nitrogen and a water bath at 37°C. Centrifugation was performed for 10 min at 10,000 g at 4°C. In accordance with the manufacturer's instructions, the supernatant is used for GSSG and GSH assays.

### 2.14 GLS1 Enzyme Activity Assay

5 × 10^5^ cells were seeded in 6‐well plates and cultured to 70%–80% confluence. After overnight attachment, the cells were treated with DMEM containing dialysed serum for 8 h. After treatment, the cells were washed twice with cold PBS to remove the medium. An appropriate volume of lysis buffer was added (as per the instructions provided by Solarbio, Cat: bc1455) to lyse the cells. The cell lysate was then subjected to high‐speed centrifugation (12,000 rpm, 4°C for 10–15 min) to remove cell debris, and the supernatant was collected for enzyme activity measurement according to the manufacturer's instructions.

### 2.15 Ammonia, Lactate and Glucose Detection

Cells (2 × 10^5^) were seeded in 12‐well plates and incubated with DMEM, and the medium was collected for ammonia, lactate and glucose detection using the VITROS 5600 Integrated System (Ortho Clinical Diagnostics) and cobas 701 (Roche).

### 2.16 Non‐Targeted Metabolomics

UHPLC–MS analysis was conducted by Novogene Co. Ltd. using a Vanquish UHPLC system with Hypesil Gold columns. The gradient of the solvent was set, and analysis was performed using a Q Exactive HF mass spectrometer. Differential metabolites were identified using Compound Discoverer 3.1 (Thermo Fisher). Metabolites meeting the criteria of VIP > 1, *p*‐value < 0.05, and fold change (FC) ≥ 2 or FC ≤ 0.5 were identified as differential metabolites through univariate analysis (*t*‐test).

### 2.17 Targeted Metabolomics

In Bioprofile Co. (Shanghai, China) Ltd., Shimadzu Nexera X2 LC‐30 ad system with Acquity UPLC HSS T3 column (1.8 μm 2.1 × 50 mm, Waters) was utilised for focused metabolomic investigation through UHPLC–MS. The ESI source parameters were set for Positive and Negative Ion Modes, and metabolites were quantified using MRM Analyser.

### 2.18 Metabolite Profiling and Isotope Tracing

LC–MS/MS analysis was conducted at the Metabolomics Facility at Tsinghua University Branch of the China National Center using a TSQ Quantiva triple quadrupole mass spectrometer and Dionex UltiMate 3000 UPLC system. For glutamine tracing, MCF‐7 and HCT116 cells were incubated with ^13^C_5_‐glutamine and glucose, extracted with methanol, and analysed using LC–MS/MS. Data were processed with Tracefinder 3.2 (Thermo). The onboarding requirements and techniques for LC–MS/MS were detailed in a previous description [6].

### 2.19 RNA‐Seq Data and Quantitative Real‐Time PCR

Total RNA was extracted and sequenced by Novogene, with cDNA libraries constructed using the VAHTS Stranded mRNA‐seq Library Prep Kit for Illumina. Libraries were sequenced using the Illumina NovaSeq 6000 platform. Data were processed using HISAT2 and Cufflinks, and differentially expressed genes were identified with DESeq2. The criteria for determining significantly differential expression were established as a *p*‐value ≤ 0.05 and an absolute log2 fold change ≥ 1.

### 2.20 Statistics

The data is presented as the average plus or minus the standard deviation. Unpaired, two‐tailed Student's *t*‐tests were utilised to compare the two groups. An asterisk in the figure denotes the presence of statistical significance (*p < 0.05; **p < 0.01; ***p < 0.001; ****p < 0.0001).

## 3 Results

### 3.1 Knockout of GLS1 Significantly Inhibited Proliferation of Cancer Cells, and Supplementation With Glutamate Rescued Cell Proliferation

**FIGURE 1:**
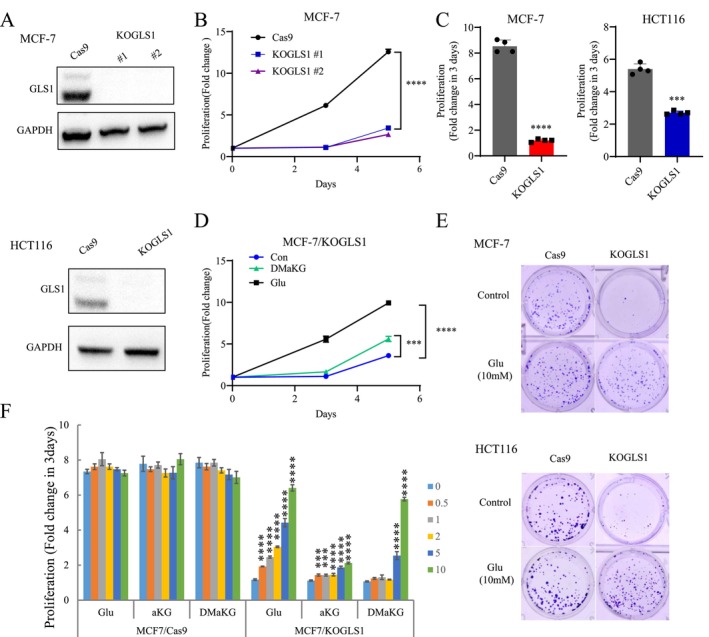
Knockout of GLS1 significantly inhibited proliferation of cancer cells, and supplementation with glutamate rescued cell proliferation. (A) WB validation of GLS1 knockout using CRISPR/Cas9 in MCF‐7 and HCT116 cells. (B, C) Cell proliferation of MCF‐7 and HCT116 cells with GLS1 knockout. (D) The cell proliferation of MCF‐7 cells with GLS1 knockout was assessed under the influence of 2 mM DMα‐KG and 10 mM glutamate supplementation in the conditioned media. (E) The colony formation assay was conducted on MCF‐7 and HCT116 cells, both with GLS1 knockout and with or without 10 mM glutamate supplementation. (F) Cell proliferation of HCT116/Cas9 and HCT116/KOGLS1 cells supplemented with different concentrations of glutamate, α‐KG and DMα‐KG (mM). All cultures were supplied with 10% dialyzed serum. Values are the means ± SEM of three independent experiments. ***p < 0.001; ****p < 0.0001 (Student's *t*‐test).

In order to investigate the role of GLS1‐regulated glutaminolysis in cancer cells, we initially utilised shRNA to knock down GLS1 in a breast cancer cell line, MCF‐7. Interestingly, the proliferation of MCF‐7 remained unaffected by either knockdown of KGA alone or both KGA and GAC (Figure S1). This led us to speculate that certain metabolic enzymes may retain their catalytic functions even at extremely low levels of expression, suggesting that knockdown of GLS1 may not inhibit its influence. Therefore, we knocked out GLS1 in breast cancer, colorectal cancer and glioma cell lines, respectively, to investigate its role. We obtained MCF‐7 and HCT116 knockout GLS1 cell lines and mainly used them as models for subsequent studies. The expression of GLS1 in these two cell lines is moderate (Figure S1), making them suitable for functional studies. In vitro proliferation assays indicated that knockout of GLS1 remarkably suppressed the proliferation of MCF‐7 and HCT116 cells (Figure 1A–C). The knockout of GLS1 resulted in gentle inhibition of ammonia secretion from MCF‐7/KOGLS1, a slight decrease in glucose consumption, and significant inhibition of lactate secretion (Figure S2A–C), suggesting potential alterations in glucose metabolism. The addition of 10 mM glutamate significantly restored the proliferation of GLS1 knockout MCF‐7 cells, and the addition of 2 mM Dimethyl α‐ketoglutarate (DMαKG) partially rescued the proliferation (Figure 1D). Clonogenic assays revealed that knockout of GLS1 vigorously repressed the clonogenic ability of MCF‐7 and HCT116 cells. Adding 10 mM glutamate substantially reversed the clonogenic ability of cancer cells (Figure 1E). In addition, knockout of GLS1 dramatically inhibited the proliferation and clone formation of the glioma cell line LN229 (Figure S2D–F). Next, we added different concentrations of α‐ketoglutarate (α‐KG), DM (Dimethyl) αKG and glutamate to detect the proliferation of MCF‐7 and HCT116 cells. To our surprise, adding high concentrations of DMαKG could rescue the proliferation inhibition in MCF‐7 cells but led to cell death in HCT116 cells (Figures 1F and S2G). A study also showed that α‐KG induces pyroptosis through caspase‐8‐mediated cleavage of Gasdermin C (GSDMC) in some types of cancer cells, including HCT116 [22]. Thus, 10 mM glutamate was essential for fully rescuing the proliferation of GLS1 knockout cells (Figures 1F and S2G), and we used this concentration in subsequent experiments.

### 3.2 Non‐Essential Amino Acids That Can Be Converted to Glutamate Can Partially Rescue the Proliferation of GLS1 Knockout Cancer Cells

**FIGURE 2:**
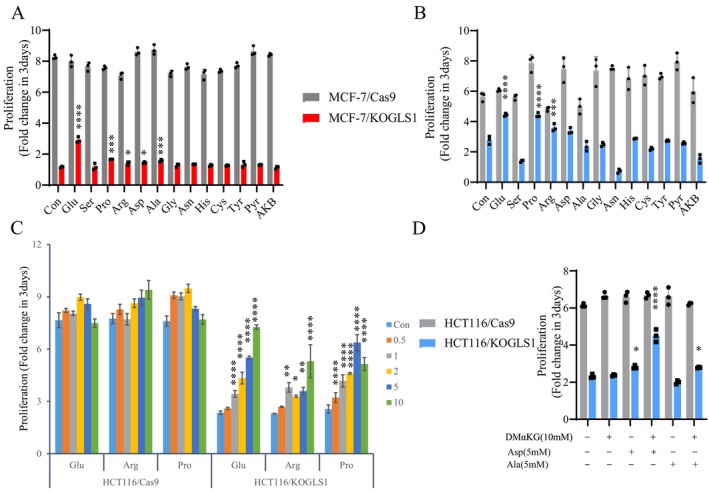
Non‐essential amino acids that can be converted to glutamate can partially rescue the proliferation of GLS1 knockout cancer cells. (A, B) Cell proliferation of GLS1 knockout in MCF‐7 and HCT116 cells and control cells supplemented with 2 mM of non‐essential amino acids, or 1 mM pyruvate and AKB. (C) The impact of varying concentrations of glutamate, arginine and proline (mM) on the cell proliferation of HCT116/Cas9 and HCT116/KOGLS1 cells. (D) Effect of adding 10 mM DMα‐KG and 5 mM aspartate or alanine on the proliferation of HCT116 cells with KOGLS1 knockout. All cultures were supplied with 10% dialysed serum. Values are the means ± SEM of three independent experiments. *p < 0.05; **p < 0.01; ***p < 0.001; ****p < 0.0001 (Student's *t*‐test).

Amino acids contribute to the accumulation of most biomass in proliferating mammalian cells [23]. Notably, the biosynthesis of non‐essential amino acids is heavily dependent on glutamine, and the GLS1‐catalysed glutaminolysis is transferred to different α‐keto acids via a family of aminotransferases to produce other Non‐Essential Amino Acids (NEAAs), which include alanine, aspartate, serine and ornithine [5]. Whether these non‐essential amino acids can be reversed to produce glutamate is not entirely determined. Eleven non‐essential amino acids other than glutamine were added to the conditioned medium to investigate whether they could rescue the proliferation of GLS1 knockout cells. In addition to glutamate, proline and arginine could also gently restore the proliferation of GLS1 knockout cells, whilst aspartate and alanine only partially rescued the proliferation of MCF‐7/KOGLS1 cells (Figures 2A,B and S3A,B). The TCA intermediate pyruvate, the electron acceptor 2‐ketobutyric acid (AKB), Branched‐Chain Amino Acids (BCAAs), and a downstream product of glutamate‐γ‐aminobutyric acid (GABA) could not rescue the proliferation of GLS1 knockout cells (Figures 2A,B and S3C,D). Subsequently, GLS1 knockout cells were employed as a model to investigate the impact of varying concentrations of arginine and proline on the proliferation of cancer cells. The findings revealed that a concentration of 10 mM of arginine, along with a concentration exceeding 0.5 mM of proline, notably enhanced the proliferation of GLS1 knockout cells, but the restorative effect on HCT116 cells was more pronounced than on MCF‐7 cells (Figures 2C and S3E). The inconsistent impact of non‐essential amino acids on the recovery of GLS1 knockout proliferation in both cell types suggests the presence of metabolic heterogeneity amongst different cancer cells. The addition of DMαKG, aspartate, or alanine alone did not result in the restoration of proliferation in HCT116/KOGLS1 cells. However, the simultaneous addition of DMαKG and aspartate significantly restored proliferation in these cells. Additionally, 10 mM DMαKG and 5 mM alanine partially restored proliferation (Figure 2D). This might be because HCT116 cells cannot utilise ammonia released by glutaminolysis, whereas breast cancer cells can utilise amide nitrogen for biosynthesis [7]. In summary, these findings suggest that the synthesis of glutamate through the reversal of proline, arginine, aspartate and alanine in cancer cells can partially reverse the proliferation inhibition caused by GLS1 knockout in MCF‐7 and HCT116 cells (Figure S3F).

### 3.3 Adding Back KGA and GAC Can Ultimately Rescue the Proliferation of GLS1 Knockout Cancer Cells

**FIGURE 3:**
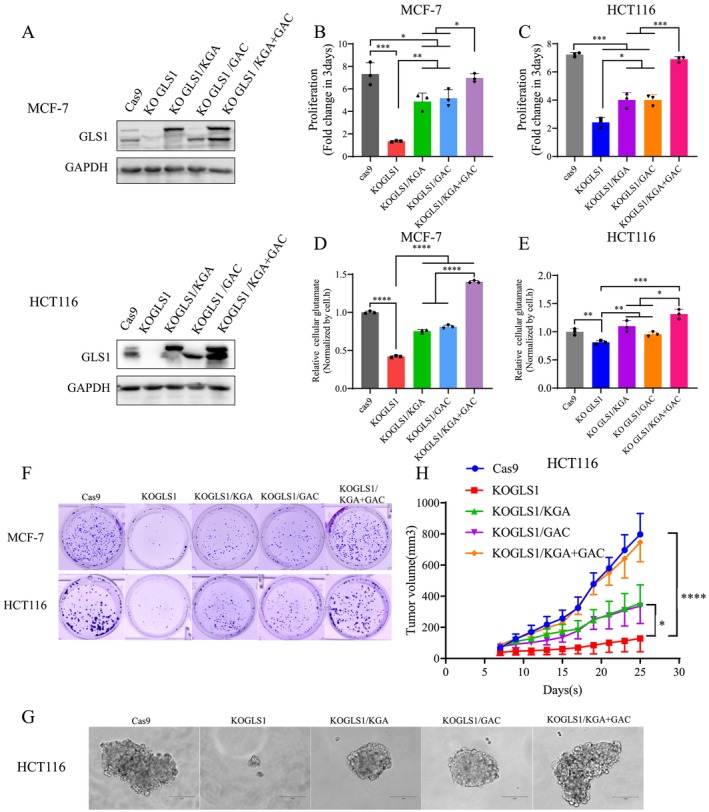
Adding back of KGA and GAC can completely rescue the proliferation of GLS1 knockout cancer cells. (A) WB validation of adding back of KGA or/and GAC in MCF‐7 and HCT116 cells with GLS1 knockout. (B, C) Influence of adding back KGA or/and GAC on the proliferation of MCF‐7/KOGLS1 and HCT116/KOGLS1 cells. (D, E) Relative abundance of cellular glutamate in Cas9, KOGLS1, KOGLS1/KGA, KOGLS1/GAC and KOGLS1/KGA + GAC cells cultured for 8 h. (F, G) Clone and sphere formation image of Cas9, KOGLS1, KOGLS1/KGA, KOGLS1/GAC and KOGLS1/KGA + GAC cells. (H) The in vivo tumour growth of HCT116/Cas9, HCT116/KOGLS1, HCT116/KOGLS1/KGA, HCT116/KOGLS1/GAC and HCT116/KOGLS1/KGA + GAC cells. All cultures were supplied with 10% dialysed serum. Values are the means ± SEM of three independent experiments. *p < 0.05; **p < 0.01; ***p < 0.001; ****p < 0.0001 (Student's *t*‐test).

To avoid the off‐target effect of CRISPR/Cas9, we added GLS1 back to GLS1 knockout cells for phenotypic study. Alternative splicing increases complexity by producing either GAC or KGA isoforms from GLS1 pre‐mRNA. The two isoforms were overexpressed separately or simultaneously into GLS1 knockout cell lines (Figure 3A). The results showed that back supplementation of GAC could largely restore the proliferation of GLS1 knockout cell lines (Figure 3B,C). Previous studies considered that the GAC spliceosome plays a more important role than KGA. To our surprise, adding back KGA also greatly rescued the proliferation inhibition caused by GLS1 knockout (Figure 3B,C). This result might explain the inability of the GLS1 inhibitor compound 968 to be applied well in the clinic because it only targets GAC [24]. Simultaneous supplementation of KGA and GAC could completely rescue the proliferation of HCT116 and MCF‐7 with GLS1 knockout (Figure 3B,C). As expected, knockout of GLS1 resulted in a significant decrease in glutamate, and the addition of KGA or GAC partially restored glutamate levels, whilst replenishment of KGA and GAC fully restored or even exceeded glutamate levels in control cells (Figure 3D,E). The clonogenicity assay and sphere‐forming experiment showed that the addition of KGA or GAC could partially restore the clonogenicity of GLS1 knockout cells. In contrast, the addition of KGA and GAC could completely rescue the number of clones (Figure 3F,G). Besides, we investigated the proliferation rate of GLS1 knockout and back‐supplemented KGA or GAC cell lines under hypoxia or impaired mitochondrial respiratory chain caused by Antimycin A (Ant A). Prolonged inhibition of the respiratory chain led to the death of all cells, whose proliferation was significantly inhibited under hypoxia but did not induce death (Figure S4A,B). Tumorigenic assays in nude mice demonstrated that knockout of GLS1 significantly inhibited tumour growth in vivo, and addition back of KGA or GAC partially rescued tumour growth, whilst simultaneous addition back completely rescued the growth inhibition caused by GLS1 knockout (Figure 3H).

### 3.4 Phospholipids Cannot Rescue the Proliferation Inhibition of Cancer Cells Caused by GLS1 Knockout

**FIGURE 4:**
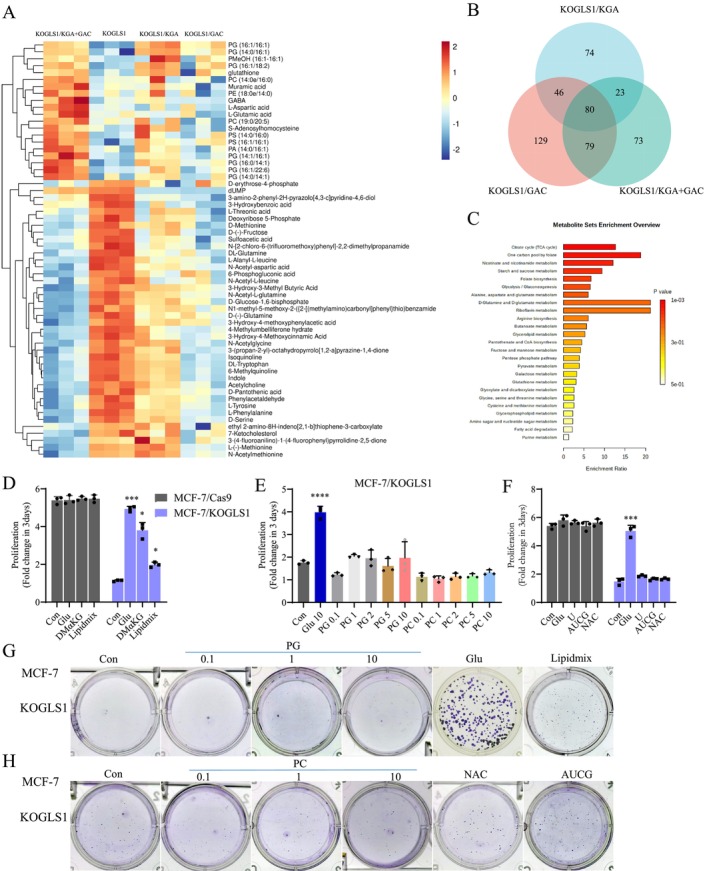
Phospholipids cannot rescue the proliferation inhibition of cancer cells caused by knockout of GLS1. (A) Heatmap of metabolites clustering in HCT116/KOGLS1/KGA + GAC, HCT116/KOGLS1, HCT116/KOGLS1/KGA and HCT116/KOGLS1/GAC cells (*p* < 0.05) cultured for 8 h measured by LC–MS‐based untargeted metabolomics. (B) Venn diagram of differential metabolites of three groups. (C) Enrichment analysis of metabolites overlapped in the Venn diagram by MetaboAnalyst 5.0. (D) Proliferation of MCF‐7/Cas9 and MCF‐7/KOGLS1 cells cultured for 3 days in the presence of glutamate (mM), DMαKG (mM), Lipid Mixture (100×). (E) Proliferation of MCF‐7/KOGLS1 cells cultured for 3 days in the presence of glutamate (mM), PG (μM), PC (μM). (F) Proliferation of MCF‐7/KOGLS1 cells cultured for 3 days in the presence of glutamate (10 mM), U (100 μM), AUCG (100 μM), or NAC (0.5 mM). (G, H) Clone formation number of MCF‐7/KOGLS1 cultured in the presence of glutamate (10 mM), PG (μM), Lipid Mixture (100 x), PC (μM), NAC (0.5 mM) or AUCG (100 μM). All cultures were supplied with 10% dialysed serum. Values are the means ± SEM of three independent experiments. *p < 0.05; ***p < 0.001; ****p < 0.0001 (Student's *t*‐test).

To identify the metabolic destination of GLS1‐catalysed glutamate generation, we performed untargeted metabolomic analysis using HCT116 cells as a model. Heat map analysis showed glutamine accumulation in the cells upon GLS1 knockout, with a significant reduction of glutamate and aspartate. Notably, several lipids, including PG and PC, were significantly decreased (Figure 4A). We performed a Venn diagram analysis of the differential metabolites of adding back of KGA or GAC alone or both GAC and KGA in GLS1 knockout cell lines and identified 80 overlapping metabolites (Figure 4B), which were analysed by metaboanalysyst and found that multiple metabolic pathways were altered: including TCA, one‐carbon unit, glutamine and glutamate metabolism, etc. (Figure 4C). The metabolite sets enrichment analysis (MSEA) network analysis of metabolites that decreased upon knockout of GLS1 revealed that glutamate metabolism, aspartate metabolism, GSH metabolism and arginine and proline metabolism were enriched (Figure S5A). Phospholipids play a crucial role in the composition of cellular membranes, with Lysophosphatidylcholine Acyltransferase 1 (LPCAT1) being responsible for regulating phospholipid saturation and oncogenic growth factor signalling. Amplification of LPCAT1 is commonly observed in cancer and is linked to unfavourable patient survival rates [25]. So, we hypothesised that the inhibition of phospholipid synthesis may lead to the cessation of proliferation in GLS1 knockout cells. We initially introduced a Lipid Mixture into the conditioned medium, which did not affect the proliferation of Cas9 cells but slightly alleviated the inhibition of proliferation in GLS1 knockout cells, although not in LN229 cells (Figures 4D,E and S5B,C). The primary constituents of phospholipids are PC and PG, and we separately incorporated various concentrations of PC and PG into the conditioned medium. PC and PG did not appear to affect Cas9 cell proliferation; however, neither PC nor PG could rescue the proliferation of GLS1 knockout cells (Figures 4E and S5D–H). Glutamine serves as a crucial precursor for nucleotide synthesis, yet the addition of Uridine (U) alone or a mixture of the four nucleosides (Adenosine, U, Cytidine, Guanosine, AUCG) failed to rescue the proliferation (Figures 4F and S5I). Besides, glutamine can also synthesise GSH to maintain redox homeostasis, and GSH levels in GLS1 knockout cells were reduced (Figure 4A). N‐acetylcysteine (NAC), as an important precursor for the synthesis of GSH, can maintain cell survival against ROS [26], but adding NAC also failed to rescue proliferation inhibition in GLS1 knockout cells (Figures 4F and S5I). Furthermore, we demonstrated that glutamate or NEAA could restore clonogenesis in GLS1 knockout cells; Lipid Mixture slightly restored, but neither PC, PG, NAC, nor AUCG rescued clonogenesis inhibition (Figures 4G,H and S6A–D). Consequently, the precise metabolic fate of glutamate catalysed by GLS1 remains to be elucidated.

### 3.5 Targeted Metabolomics and Transcriptomics Analysis of Metabolic and Gene Regulatory Changes Upon GLS1 Knockout

**FIGURE 5:**
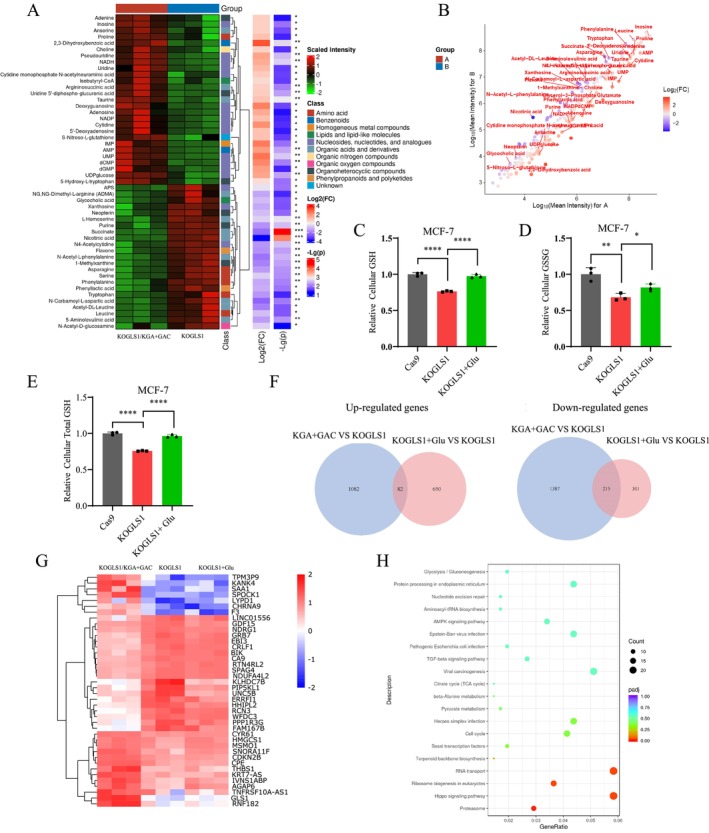
Targeted metabolomics and transcriptomics analysis of metabolic and gene regulatory changes upon GLS1 knockout. (A, B) Heatmap of metabolites clustering and scatterplot analysis of MCF‐7/KOGLS1/KGA + GAC and MCF‐7/KOGLS1 cultured for 8 h measured by LC–MS‐based targeted metabolomics. (C–E) Intracellular GSH, GSSG and total GSH were measured in MCF‐7/Cas9, MCF‐7/KOGLS1 and MCF‐7/KOGLS1 + glutamate (10 mM) cells. (F) Venn diagram analysis of differentially transcribed genes in MCF‐7/Cas9 vs. MCF‐7/KOGLS1 and MCF‐7/KOGLS1 + glutamate (10 mM) vs. MCF‐7/KOGLS1 cells. (G) Heatmap analysis of transcriptional difference genes (top 20 elevated or decreased) in MCF‐7/Cas9, MCF‐7/KOGLS1 and MCF‐7/KOGLS1 + glutamate (10 mM) cells. (H) KEGG enrichment was analysed for genes that were elevated in MCF‐7/KOGLS1/KGA + GAC compared to MCF‐7/KOGLS1 cells. All cultures were supplied with 10% dialyzed serum. Values are the means ± SEM of three independent experiments. *p < 0.05; **p < 0.01; ***p < 0.001; ****p < 0.0001 (Student's *t*‐test).

To further characterise the metabolic fate of glutamate catalysed by GLS1, we used MCF‐7/KOGLS1 cells as a model and performed targeted metabolomic analysis. The results showed that several nucleotides, including inosine, adenine, inosine monophosphate (IMP), U, C, A, UMP, AMP, deoxycytidine‐monophosphate (dCMP), and dGMP, were significantly decreased in GLS1 knockout cell lines (Figure 5A,B). However, these nucleotides were not significantly upregulated by the addition of glutamate (Figure S7A), further suggesting that the decrease in nucleotides was probably a side effect of slowed cell proliferation. Knockout of GLS1 resulted in a decrease of glutamate, proline and arginine and an increase of asparagine, ornithine, leucine and serine, but they were not reversed by glutamate supplementation (Figure S7B–H). We analysed the alteration of metabolites after glutamate supplementation in GLS1 knockout cells and discovered a significant rise of GSSG and S‐Nitrosoglutathione (Figure S7I), implying that GSH metabolism may be altered. To verify this result, we examined the changes of GSH, GSSG and total GSH and observed a significant decrease in GSH, GSSG, and total GSH after knockout of GLS1 and recovery of their levels after supplementation of glutamate (Figures 5C–E and S8A–C), suggesting that glutamate may synthesise GSH to play a role in boosting cancer cell proliferation. However, these findings are incongruent with our previous findings that NAC was unable to reverse the inhibitory effects on proliferation caused by GLS1 knockout. Our meticulous examination uncovered that, apart from cysteine, glutamate itself is an obligatory substrate for the synthesis of GSH. Consequently, we intend to investigate and substantiate this hypothesis thoroughly. The analysis of transcriptomic data demonstrated that knockout of GLS1 significantly impacted mRNA expression levels of various genes, resulting in a notable decrease in GLS1 mRNA. However, no changes were observed in mRNA levels of metabolic enzymes involved in GSH synthesis (Figures 5F,G and S8D–I). Furthermore, the KEGG pathway analysis indicated that the enrichment of the AMPK signalling pathway was observed upon expression of KGA + GAC or glutamate supplementation (Figures 5H and S8J). This finding suggests that the AMPK signalling pathway may possess a crucial regulatory function. In summary, the above results indicate that GSH synthesis was reduced in cancer cells upon GLS1 knockout, and GSH metabolism could be restored after glutamate supplementation.

### 3.6 GLS1 Knockout Leads to a Notable Decrease in the Metabolic Flux of Glutamine Towards GSH

**FIGURE 6:**
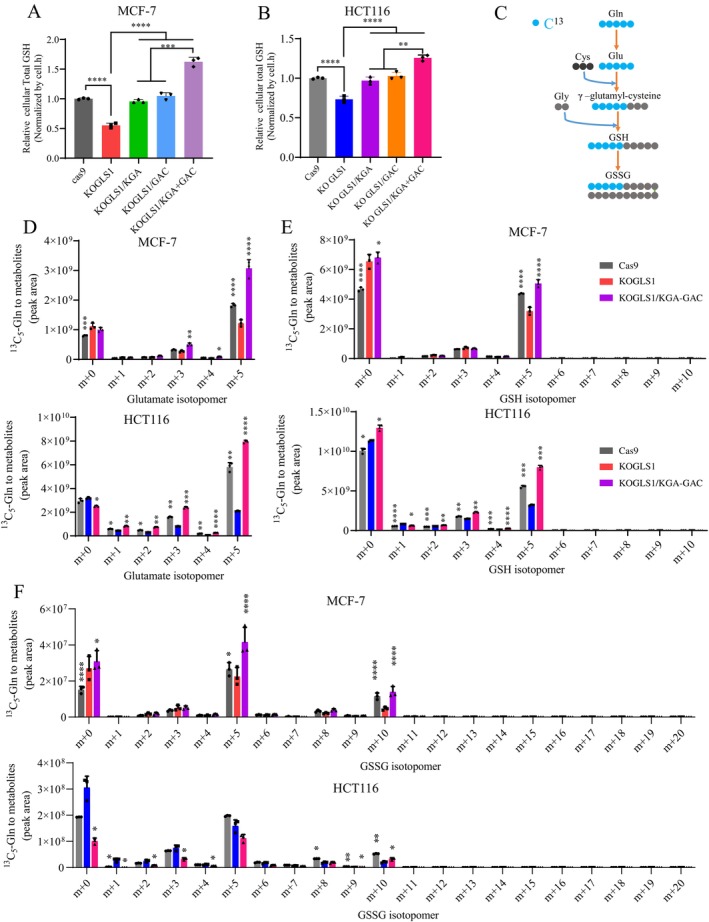
GLS1 knockout leads to a notable decrease in the metabolic flux of glutamine towards GSH. (A, B) Relative abundance of total GSH in Cas9, KOGLS1, KOGLS1/KGA, KOGLS1/GAC and KOGLS1/KGA + GAC cells cultured for 8 h. (C) A schematic to show the metabolism of isotope‐labelled glutamine to GSH. (D–F) Mass isotopomer analysis of glutamate, GSH and GSSG in Cas9, KOGLS1 and KOGLS1/KGA + GAC cultured with the medium containing 1 mM of ^13^C_5_‐glutamine for 8 h. All cultures were supplied with 10% dialyzed serum. Values are the means ± SEM of three independent experiments. *p < 0.05; **p < 0.01; ***p < 0.001; ****p < 0.0001 (compared with KOGLS1) (Student's *t*‐test).

To ascertain the relationship between GSH metabolism and glutamate production from GLS1‐catalysed glutaminolysis, we measured GSH levels in cells. The results indicated that GSH levels in MCF‐7 and HCT116 cells were reduced upon GLS1 knockout and that intracellular GSH levels were restored by adding back KGA or GAC. In contrast, re‐supplementation with KGA and GAC led to a further increase in GSH. (Figures 6A,B and S9A,B). Next, we traced the assimilation of glutamine carbon using ^13^C_5_‐glutamine. Analysis of metabolic mass spectrometry data revealed that knockout of GLS1 led to an accumulation of glutamine and a reduction in glutamate levels. Adding back KGA + GAC effectively reversed this phenomenon (Figure S9C,D). The metabolic pathway of glutamine leading to the production of GSH was schematically delineated. Specifically, the utilisation of five carbon‐labelled glutamine resulted in the generation of five carbon‐labelled glutamate, which subsequently gave rise to five carbon‐labelled GSH and either five or ten carbon‐labelled GSSG (Figure 6C). The findings from the metabolic flux analysis indicate that a substantial proportion (over 80%) of intracellular glutamine originates from external sources, whilst approximately 60% of glutamate is labelled, suggesting the existence of alternative pathways for glutamate synthesis in cancer cells (Figures 6D and S9E, Tables [Link], [Link]). Notably, knockout of GLS1 led to a significant decrease in labelled glutamate; however, it did not entirely impede glutamate production from glutamine, possibly due to the presence of an alternative pathway facilitated by Carbamoyl‐phosphate synthetase II, Aspartate transcarbamylase and Dihydroorotase (CAD). Consistently, adding back KGA and GAC further increased the metabolic flux of glutamine to glutamate (*m* + 5) (Figures 6D and S9E, Tables [Link], [Link]). 70% of γ‐glutamylcysteine was derived from glutamine (about 30% unlabeled) in MCF‐7 and HCT116 cells (Tables [Link], [Link]). Nearly 70% of GSH and GSSG were derived from glutamine (30% unlabeled) in MCF‐7 cells, and about 50% of GSH and 70% of GSSG were derived from glutamine in HCT116 cells (Figure 6E,F, Tables [Link], [Link]). Knockout of GLS1 resulted in a significant decrease in glutamine to γ‐glutamylcysteine *m* + 5, GSH *m* + 5, and GSSG *m* + 5 or *m* + 10, which was reversed by adding back KGA + GAC (Figures 6E,F and S9F, Tables [Link], [Link]). These findings suggest that GLS1 knockout effectively diminishes the metabolic flux of glutamine towards GSH.

### 3.7 GSH Rescues Proliferation Depression of Cancer Cells Owing to GLS1 Knockout

**FIGURE 7:**
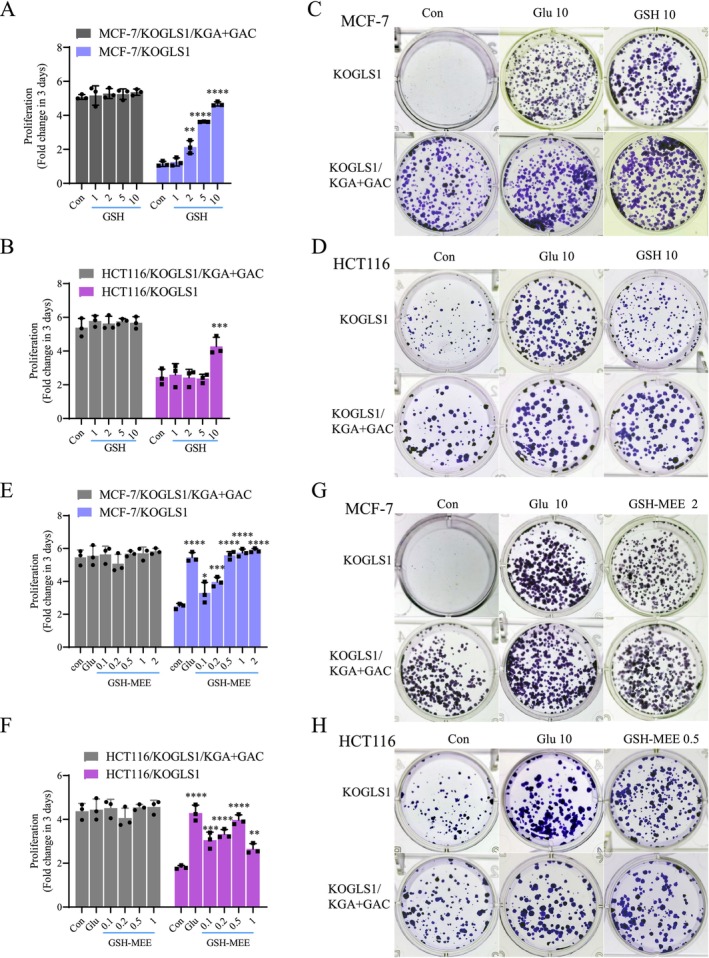
GSH rescues proliferation depression of cancer cells owing to GLS1 knockout. (A, B) Proliferation of KOGLS1/KGA + GAC and KOGLS1 cells after addition of GSH (mM) to conditioned medium. (C, D) Clone formation by adding glutamate or GSH to conditioned medium in KOGLS1 and KOGLS1/KAG + GAC cells. (E, F) Proliferation of KOGLS1/KGA + GAC and KOGLS1 cells by adding glutamate or GSH‐MEE (mM) to conditioned medium. (G, H) Clone formation by adding glutamate or GSH‐MEE (mM) to conditioned medium of KOGLS1 and KOGLS1/KAG + GAC cells. All cultures were supplied with 10% dialysed serum. Values are the means ± SEM of three independent experiments. *p < 0.05; **p < 0.01; ***p < 0.001; ****p < 0.0001 (Student's *t*‐test).

We then added different concentrations of GSH to the conditioned medium and investigated its proliferative rescue effect on GLS1 knockout cells. Since we were unsure about the ability of GSH to enter into the cells, we added different concentrations of GSH to the conditioned medium. 10 mM GSH fully restored MCF‐7/KOGLS1 cell proliferation and largely restored HCT116/KOGLS1 cell proliferation, with no significant impact on control cell proliferation (Figure 7A,B). Clonogenic assays showed similar results (Figure 7C,D). Further, we used GSH monoethyl ester (GSH‐MEE), which is hydrolyzed by intracellular esterases to release GSH, thereby increasing the concentrations of intracellular GSH, to confirm its ability to facilitate the proliferation of GLS1 knockout cells. 0.5 mM GSH‐MEE was able to rescue the proliferation of MCF‐7/KOGLS1 and HCT116/KOGLS1 cells (Figure 7E,F). 2 mM GSH‐MEE was able to restore MCF‐7/KOGLS1 clonogenic capacity, and HCT116/KOGLS1 cells required only 0.5 mM GSH‐MEE to restore clonogenic capacity, without significant impact on control cells (Figures 7G,H and S10A). To investigate whether intracellular glutamate is preferentially used to exchange cystine into the cell via xCT or for biosynthesis, we detected glutamate in the cell culture medium after GLS1 knockout and found that the knockout of GLS1 resulted in reduced glutamate in the culture medium, but the adding back of either KGA or GAC restored glutamate secretion (Figure S10B,C), suggesting that intracellular glutamate synthesised by KGA or GAC in cancer cells may first be used to exchange cystine for GSH synthesis.

### 3.8 Knockout of GLS1 Leads to Impaired Antioxidant Capacity and Increased Ferroptosis Sensitivity in Cancer Cells

**FIGURE 8:**
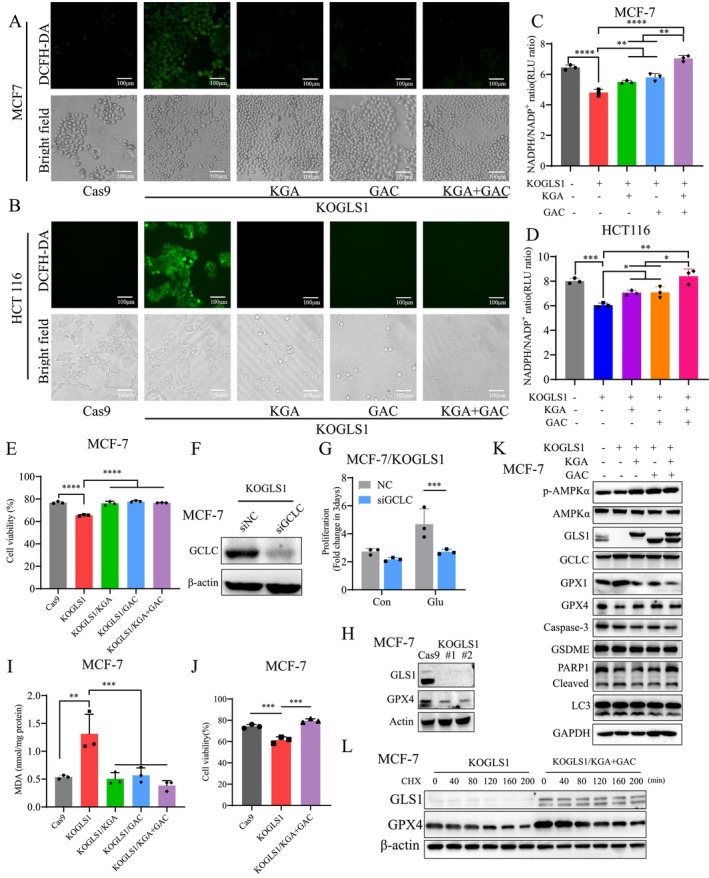
Knockout of GLS1 leads to impaired antioxidant capacity and increased ferroptosis sensitivity in cancer cells. (A, B) Detection of the intracellular ROS levels in Cas9, KOGLS1, KOGLS1/KGA, KOGLS1/GAC and KOGLS1/KGA + GAC cells. (C, D) NADPH/NADP^+^ ratio was measured in Cas9, KOGLS1, KOGLS1/KGA, KOGLS1/GAC and KOGLS1/KGA + GAC cells. (E) Relative cell death after the addition of 50 μM H_2_O_2_ to conditioned media for 4 h. (F, G) Proliferation of MCF‐7/KOGLS1 cells with down‐regulated GCLC was detected after the addition of 10 mM glutamate to the conditioned medium. (H) Detection of GPX4 expression in MCF‐7 with GLS1 knockout cells using Western blot. (I) Relative abundance of MDA in MCF‐7/Cas9, MCF‐7/KOGLS1, MCF‐7/KOGLS1/KGA, MCF‐7/KOGLS1/GAC and MCF‐7/KOGLS1/KGA + GAC cells. (J) Relative cell death after the addition of 5 μM erastin to conditioned media for 24 h. (K) Protein expressions were detected in MCF‐7/Cas9, MCF‐7/KOGLS1, MCF‐7/KOGLS1/KGA, MCF‐7/KOGLS1/GAC and MCF‐7/KOGLS1/KGA + GAC cells by Western blot. (L) 10 mg/mL CHX was added, with protein samples collected at different time points to examine GPX4 protein stability via Western blot. All cultures were supplied with 10% dialyzed serum. Values are the means ± SEM of three independent experiments. *p < 0.05; **p < 0.01; ***p < 0.001; ****p < 0.0001 (Student's *t*‐test).

GSH is essential for scavenging intracellular ROS and maintaining oxidative homeostasis [27]. We first examined ROS levels in GLS1 knockout cells. The intracellular ROS significantly increased upon GLS1 knockout, and reintroduction of KGA, GAC, or both KGA and GAC reduced intracellular ROS levels (Figure 8A,B). To further confirm the correlation between GLS1 knockout‐induced ROS accumulation and tumour growth, we utilised Cas9, KOGLS1 cells, and positive control of 50 μM hydrogen peroxide (H_2_O_2_). Notably, KOGLS1 cells exhibited ROS levels exceeding those induced by 50 μM H_2_O_2_, a concentration sufficient to inhibit tumour cell proliferation (Figure S11A). NADPH, responsible for maintaining GSH in its reduced state and acting as a crucial coenzyme for GSH reductase [28], was also reduced upon GLS1 knockout. This reduction was largely reinstated by KGA or GAC and fully restored by the adding back of KGA and GAC (Figure 8C,D). Propidium iodide and Hoechst double staining demonstrated that GLS1 knockout heightened the sensitivity of MCF‐7 and HCT116 cells to H_2_O_2_ (Figures 8E and S11B). Additionally, downregulation of glutamate‐cysteine ligase catalytic subunit (GCLC), a key enzyme in GSH synthesis, showed that glutamate addition did not rescue the proliferation of GLS1 knockout cells, indicating glutamate's role in GSH synthesis is crucial for cell proliferation (Figures 8F,G and S11C,D). GSH, a non‐enzymatic antioxidant system, is closely linked to the occurrence of ferroptosis, a form of cell death driven by ROS [29, 30]. Notably, GLS1 knockout led to a significant decrease in GPX4 expression, a key ferroptosis regulator (Figure 8H). The accumulation of malondialdehyde (MDA), an indicator of lipid peroxidation, was mitigated by reintroduction of KGA, GAC, or both (Figures 8I and S11E). Erastin, a ferroptosis inducer [31], caused a higher percentage of cancer cell death in GLS1 knockout cells, which was rescued by KGA and GAC (Figures 8J and S11F). These findings suggest that GLS1 knockout enhances the susceptibility of cancer cells to ferroptosis. Western blot analysis showed that GLS1 knockout downregulated GPX4 expression in MCF‐7 and HCT116 cells, which was restored by KGA, GAC, or both, without significantly upregulating markers for apoptosis, autophagy, pyroptosis and DNA damage repair (Figures 8K and S12A,B). GLS1 knockout also increased the expression of GCLC, likely due to decreased glutamate production triggering reactive upregulation, which was counteracted by KGA, GAC, or both (Figures 8K and S12A,B). Additionally, GPX1 expression, another GPX family member, increased following GLS1 knockout and reverted upon KGA and GAC reintroduction (Figure 8K and S12A,B). Furthermore, consistent with the transcriptomics results, GLS1 knockout decreased AMPK phosphorylation, which was reversed by KGA, GAC, or both (Figures 5H, 8K, and S8J, S12A–C). Additionally, GLS1 inhibition with BPTES also induces AMPK dephosphorylation (Figure S12D), suggesting that AMPK may sense changes in GLS1 or the metabolites it influences to regulate cellular energy balance. Previous studies by Lee et al. and Yang et al. also showed that AMPK inactivation sensitises cancer cells to ferroptosis [32, 33]. Upon H_2_O_2_ stimulation, GPX4 expression increased whilst GPX1 decreased (Figure S12E,F), suggesting complementary roles for GPX4 and GPX1 in cancer cells. Since GLS1 knockout did not result in significant changes in GPX4 mRNA (Figure S8G), we treated GLS1 knockout and KGA + GAC rescue cells with cycloheximide (CHX) to study how GLS1 regulates GPX4 expression. Our results indicate that knockout of GLS1 accelerates the degradation rate of GPX4 (Figures 8L and S12G), suggesting that GLS1 plays a role in stabilising GPX4 expression. In summary, these results suggest that glutamate generated by GLS1‐catalysed glutaminolysis primarily supports GSH biosynthesis. Knockout of GLS1 leads to increased ROS and MDA levels, decreased NADPH/NADP^+^ ratio, and heightened cell sensitivity to ferroptosis.

### 3.9 GLS1, GPX4 and GPX1 Inhibitors Synergistically Suppress Cancer Cell Growth

**FIGURE 9:**
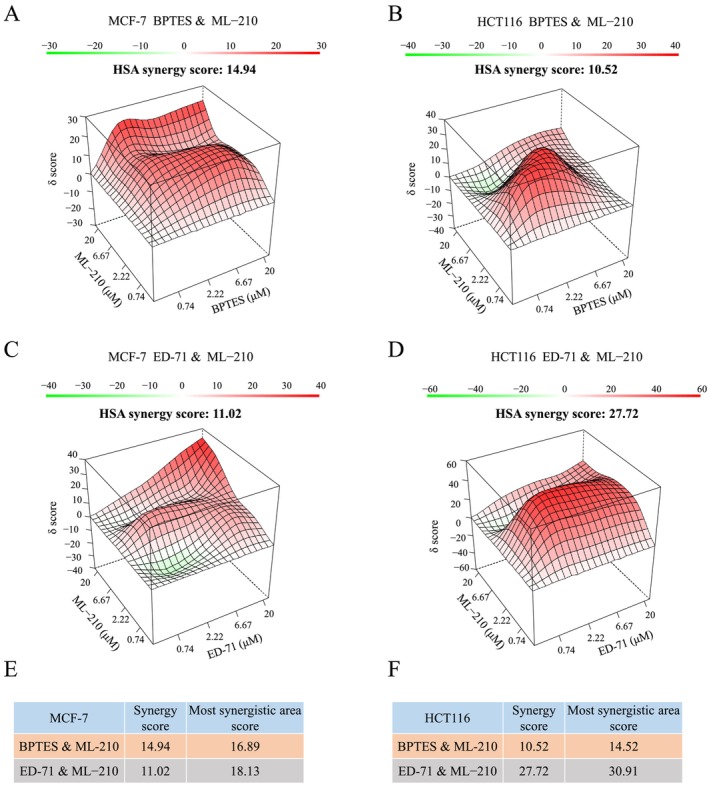
GLS1, GPX4 and GPX1 inhibitors synergistically suppress cancer cell growth. (A, B) Synergy analysis of BPTES and ML‐210 at different concentrations in MCF‐7 and HCT116 cells after 72 h of co‐treatment, calculated using the Synergy Finder online tool (https://synergyfinder.fimm.fi/synergy). (C, D) Synergy analysis of ED‐71 and ML‐210 at different concentrations in MCF‐7 and HCT116 cells after 72 h of co‐treatment, using Synergy Finder. (E, F) Summary of synergy scores and the highest synergy region index for different drug combinations. All cultures were supplied with 10% dialysed serum. Values are the means ± SEM of three independent experiments.

Cancer cells can rewire metabolic pathways, leading to resistance, which is a major reason why many metabolic enzyme inhibitors fail to exhibit anti‐cancer effects. In our study, we found that GLS1 knockout increases cancer cell sensitivity to ferroptosis by downregulating GPX4. However, we also observed an upregulation of GPX1, allowing the cells to survive and proliferate. To address this, we explored whether simultaneous inhibition of GLS1, GPX4 and GPX1 could synergistically inhibit cancer cell growth. We conducted experiments using the GLS1 inhibitor BPTES. First, we confirmed that GLS1 knockout significantly reduced glutaminase activity, though some residual activity remained. We hypothesise that GLS2 might compensate for this reduction, partially restoring activity. Additionally, restoring both splice variants could lead to even stronger activity (Figure S13A). The GLS1 inhibitor BPTES can effectively suppress its enzymatic activity (Figure S13B). Next, using MCF‐7 and HCT116 cells as models, we performed combination drug treatments with GLS1 inhibitor BPTES, GPX4 inhibitor ML‐210, and GPX1 inhibitor Eldecalcitol (ED‐71). After determining the Half‐Maximal Inhibitory Concentration (IC50) values of each drug (Figure S13C–H), we combined the drugs in a 4 × 4 concentration matrix to assess their potential synergistic effects on MCF‐7 and HCT116 cell growth. We quantified the synergy using the HSA Synergy Score, and the results showed that the combination of BPTES and ML‐210 exhibited significant synergistic effects in inhibiting cancer cell growth (synergy index > 10) (Figure 9A,B,E,F). This suggests that inhibiting GLS1 can synergize with GPX4 inhibitors (which promote ferroptosis) to effectively suppress tumour growth. Furthermore, the combination of ML‐210 and ED‐71 also synergistically inhibited tumour growth (synergy index > 10) (Figure 9C–F), as the simultaneous inhibition of GPX4 and GPX1 disrupts their compensatory responses, offering a promising strategy for cancer therapy.

### 3.10 GLS1 and GPX4 Are Highly Expressed in Cancer Patient Tissues and Correlated With Poor Prognosis

**FIGURE 10:**
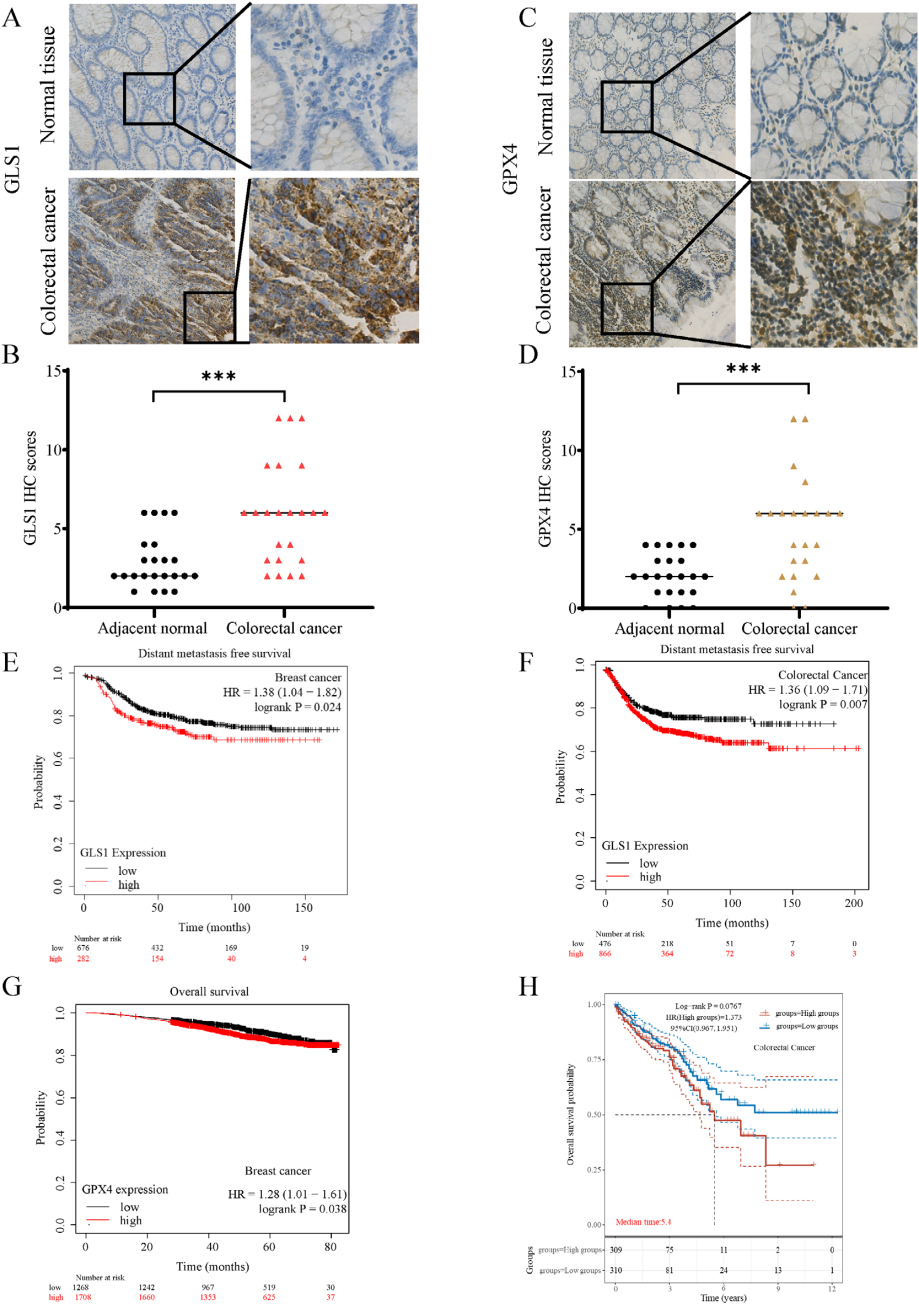
GLS1 and GPX4 are highly expressed in cancer patient tissues and correlated with poor prognosis. (A, B) Expression of GLS1 in 23 paired cancer and paracancer tissues of colorectal cancer patients was detected by immunohistochemistry. (C, D) Expression of GPX4 in 23 paired cancer and paracancer tissues of colorectal cancer patients was detected by immunohistochemistry. (E–H) The survival analysis was performed on the Kaplan–Meier plotter database (https://www.kmplot.com/) and ASSISTANT for Clinical Bioinformatics (https://www.aclbi.com/static/index.html#/prognosis). ***p < 0.001 (Student's *t*‐test).

To confirm this metabolic phenomenon in cancer patients, we obtained cancerous and paracancerous tissue samples from 23 pairs of colorectal cancer patients and examined GLS1 and GPX4 expression through immunohistochemistry. Our findings showed a significant increase in GLS1 expression in cancerous tissues compared to adjacent paracancerous tissues (Figure 10A,B), indicating enhanced glutaminolysis in tumour tissues. Similarly, GPX4 expression was significantly higher in cancerous tissues than in paracancerous tissues (Figure 10C,D). These results suggest maintaining redox balance is essential for tumour growth in vivo. Significantly, the analysis of online data revealed that patients with colorectal and breast cancer who exhibited high expression of GLS1 experienced a shorter duration of Distant Metastasis Free Survival (DMFS) compared to those with low expression, which suggests that GLS1 plays a crucial role in influencing the prognosis of colorectal and breast cancer patients (Figure 10E,F). Besides, individuals with colorectal cancer who had high GLS1 expression demonstrated a reduced overall survival, and breast cancer patients with high GLS1 expression experienced a shorter Recurrence Free Survival (RFS) (Figure S14A,B). Furthermore, breast and colorectal cancer patients with high expression of GPX4 have poor overall survival rates (Figure 10G,H). Overall, the data above indicate that GLS1 and GPX4 show high levels of expression in cancer patients' tissues and are linked to unfavourable outcomes.

## 4 Discussion

The potential impact of metabolic changes on tumorigenesis and cell survival has been widely recognised in cancer research [34, 35]. Glutamine, an essential nutrient for cancer cells, has been discovered to have a major impact on aiding the production of different amino acids and nucleotides, ultimately promoting the growth and spread of cancer cells [36, 37]. Furthermore, glutamine has been shown to provide energy and inhibit the accumulation of reactive oxygen species (ROS) in cancer cells [17, 38]. Clinical trials investigating inhibitors of GLS1, the enzyme responsible for glutaminolysis, are currently underway [4]. However, the precise involvement of glutamate derived from glutaminolysis in the metabolic processes of cancer cells remains incompletely understood. In our study, we found that silencing KGA and/or GAC did not impede cancer cell growth, whereas GLS1 knockout significantly inhibited cancer proliferation. Exogenous supplementation with glutamate or non‐essential amino acids that can convert to glutamate fully/partially restored proliferation inhibition in GLS1 knockout cell lines. In vitro and in vivo experiments showed that simultaneous addition of KGA and GAC completely rescued rapid cancer cell proliferation. Various assays demonstrated that glutamate generated by glutaminolysis is primarily used for GSH synthesis, and exogenous GSH or GSH‐MEE can rescue proliferation inhibition caused by GLS1 knockout. Cancer cell sensitivity to ferroptosis increased upon GLS1 knockout. GPX4 and GPX1 function complementarily in redox regulation, with GLS1 knockout promoting GPX4 degradation. Inhibiting GLS1 enhances the efficacy of ferroptosis inhibitors in suppressing tumour growth, and simultaneously targeting GPX4 and GPX1 offers a powerful anti‐cancer approach. Notably, both GLS1 and GPX4 exhibit high expression levels in tumour tissues, which are associated with an unfavourable prognosis.

Certain metabolic enzymes can catalyse reactions efficiently even with low expression [39], limiting shRNA technology's application in cancer metabolism studies. Our study showed that GLS1 downregulation in MCF‐7 cells did not effectively impede proliferation, whereas GLS1 knockout significantly inhibited proliferation and clone formation in various cancer cells, demonstrating CRISPR technology's superiority in studying metabolic enzyme functionality. Adding proline, arginine, aspartate, or alanine to the conditioned medium restored GLS1 knockout cell proliferation, suggesting bidirectional interconversion amongst these amino acids. This discovery establishes a theoretical basis for exploring potential synergistic applications. Whilst the expression levels of spliceosomal KGA and GAC may vary in cancer cells, both enzymes convert glutamine to glutamate. Empirical investigations demonstrated that reintroducing KGA or GAC partially reinstates proliferation in GLS1 knockout cell lines, whilst both KGA and GAC fully rescue proliferation, indicating their substantial influence on cancer cells. This may explain the limited use of compound 968, which selectively targets spliceosomal GAC, in cancer treatment [24].

Glutamine plays a vital role in metabolic processes, serving as a nitrogen and carbon source in biosynthetic pathways and is essential for energy generation [40]. Our study's metabolomics and cell proliferation assays show that the reduction in intracellular metabolites (e.g., nucleotides, GABA, phospholipids) observed in GLS1 knockout is due to suppressed cell proliferation rather than the cause. Exogenous supplementation of these metabolites fails to restore cancer cell proliferation, but GSH or GSH‐MEE addition successfully reverses suppression. Additionally, the metabolic flux of glutamine to GSH is reduced in GLS1 knockout cells, and exogenous glutamate restores intracellular GSH levels. Downregulation of GCLC, a key enzyme in GSH synthesis, prevents glutamate from rescuing GLS1 knockout cells, suggesting glutamate's significant role in promoting cell proliferation through GSH synthesis. A Pan‐Cancer study confirms redox homeostasis as a critical function of GLS1 in tumours [41].

Unlike apoptosis, necrosis and autophagy, ferroptosis is an iron‐dependent form of programmed cell death [42]. When divalent iron or ester oxygenase is present, ferroptosis is caused by catalysing the peroxidation of highly expressed unsaturated fatty acids on the cell membrane; in addition, it is also manifested by a decrease in the regulatory core enzyme GPX4 of the antioxidant system (GSH system) [43]. Studies have shown that MBOAT1/2 suppresses ferroptosis through phospholipid remodelling independently of GPX4 [44]. In our investigation, MBOAT1/2 mRNA expression did not change upon knockout of GLS1(S7H‐7I); the disruption of GLS1 resulted in the stimulation of intracellular ROS and MDA accumulation whilst concurrently diminishing the NADPH/NADP^+^ ratio. Consequently, this led to a decline in antioxidant capacity and an augmented vulnerability to ferroptosis. Importantly, knockout of GLS1 led to a significant decrease in GPX4 expression without concomitant upregulation of other cell death markers. Reintroduction of KGA, GAC, or KGA + GAC effectively restored GPX4 expression levels. These findings further imply that the primary function of GLS1‐mediated glutamate generation is to synthesise GSH in order to uphold redox homeostasis against ferroptosis. Cancer cells often reprogram metabolic pathways to develop drug resistance. Our findings demonstrate that targeting GLS1 and GPX4 synergistically inhibits cancer cell growth, suggesting that GLS1 inhibition can sensitise tumour cells to ferroptosis. Furthermore, our research reveals that GPX4 and GPX1 play complementary roles in defending against ferroptosis, providing a theoretical foundation for novel combination therapies. We also show that dual inhibition of GPX4 and GPX1 synergistically inhibits cancer cell growth, validating the effectiveness of targeting this compensatory metabolic mechanism. This underscores the potential of dual inhibition strategies as a promising approach for cancer treatment.

AMPK, a pivotal molecule in regulating cellular energy metabolism, can be activated by a diverse range of stimuli, such as cellular stress, exercise and various hormones and substances that influence cellular metabolism [45]. Previous studies have shown that AMPK activation helps protect against ferroptosis [32]. AMPK activates GLS1 by phosphorylating threonine 527 (T527) of PDZ domain containing 8 (PDZD8), promoting the interaction between PDZD8 and GLS1 [46]. Besides, activated AMPK enhances GLS1 stability via mitochondrial heat shock protein 75 (HSP75) [47]. Our results indicate that the depletion of GLS1 leads to a reduction in AMPK activity, and the reintroduction of KGA or GAC restores AMPK activity, suggesting a potential regulatory role of AMPK in ferroptosis in response to GLS1‐mediated glutamine catabolism. We speculate that the alterations in metabolites (e.g., glutamate, GSH, NADPH/NADP^+^) or energy status caused by GLS1 knockout lead to the dephosphorylation of AMPK. However, as a powerful energy regulator, the activation and inactivation of AMPK is a dynamic process. We will further explore the underlying mechanism of AMPK dephosphorylation by GLS1 knockout in the coming study.

**FIGURE 11:**
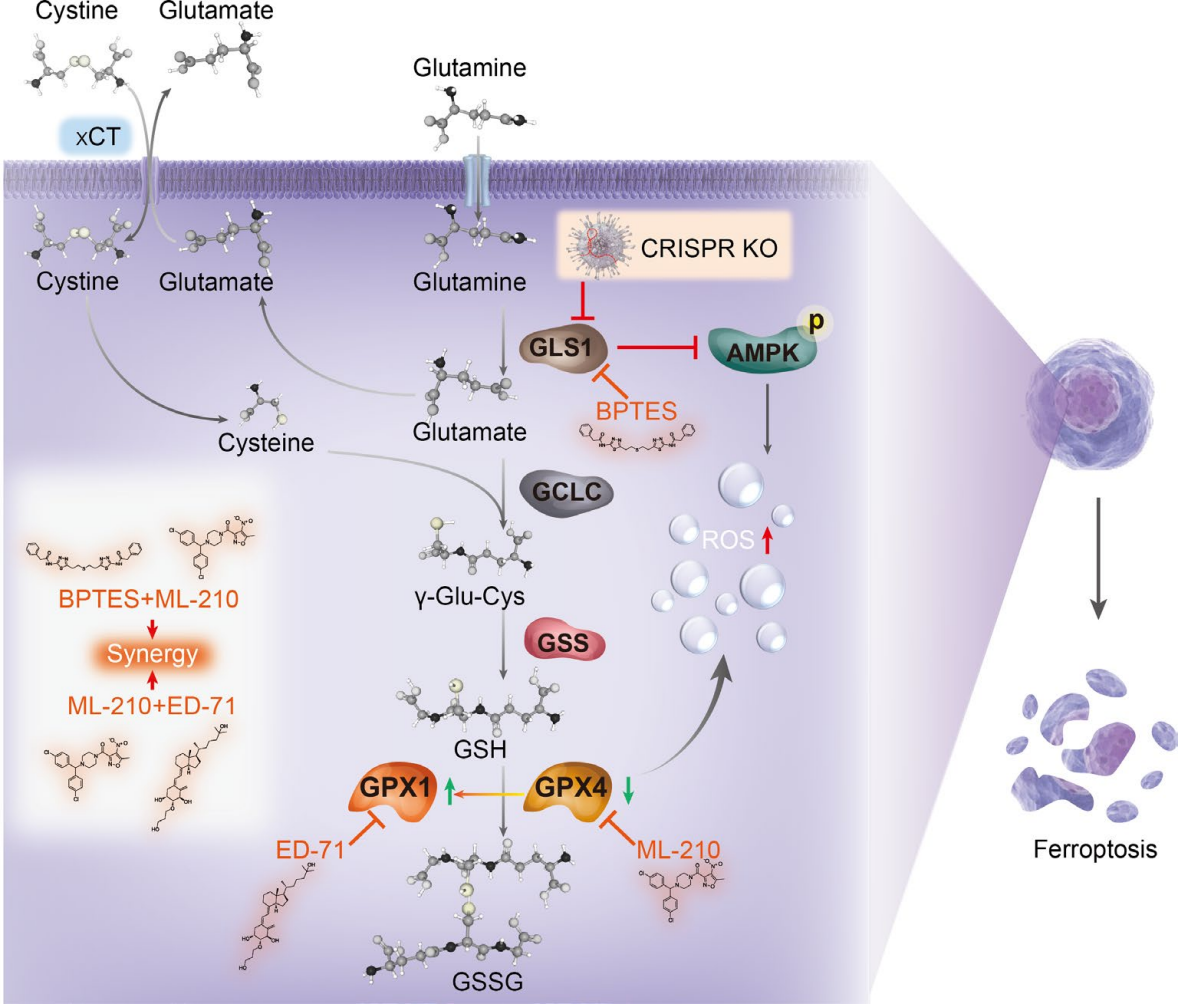
Schematic diagram of GLS1‐mediated glutaminolysis to GSH synthesis maintains redox homeostasis and modulates ferroptosis sensitivity in cancer cells. The primary metabolic outcome of glutamate generated via glutaminolysis mediated by GLS1 in cancer cells is to support the synthesis of GSH. Removal of GLS1 leads to an increase in ROS and an enhanced vulnerability of cells to ferroptosis. GLS1 knockout results in the downregulation of GPX4 but the upregulation of GPX1. Combining a GLS1 inhibitor with a GPX4 inhibitor, or a GPX4 inhibitor with a GPX1 inhibitor, synergistically suppresses cancer cell growth. Additionally, GLS1 suppression leads to a reduction in AMPK activity, which may further enhance the sensitivity of cancer cells to ferroptosis.

In conclusion, our study has provided evidence for the significant involvement of glutamate, produced through GLS1‐mediated glutaminolysis, in promoting cancer cell growth. The primary function of glutamate is to facilitate the biosynthesis of GSH, maintain intracellular redox homeostasis, and modulate the susceptibility of cancer cells to ferroptosis. GPX4 and GPX1 exhibit complementary functions in this process; targeting GLS1 in combination with GPX4 synergistically enhances the anticancer effect, and inhibiting GPX4 and GPX1 also shows synergistic effects. GLS1 knockout suppresses the AMPK signaling pathway, potentially further impairing the ability of cancer cells to counteract ROS (Figure 11). Additionally, we have identified the indispensable role of the splice variants KGA and GAC in cancer cell proliferation. This metabolic mechanism facilitates a deeper comprehension of the abnormal glutamine metabolism in cancer cells, establishing a theoretical basis for the potential clinical utilisation of GLS1 inhibitors and offering novel perspectives for advancing combination targets.

## Author Contributions

C.B., Y.L., Y.Li, X.C., and L.L. contributed to the conception and design of the study protocol. C.B., J.H., D.M., Y.X., and Z.W. performed cell and animal experiments. Y.X., B.Z., M.L., and L.L. performed immunohistochemistry and analysed metabolomics data. W.Z. and H.W. analysed transcriptomics data and bioinformatics data. C.B. wrote and revised the manuscript. Y.L., L.L., X.C., and Y.L. supervised the research process. All authors had access to the raw data and had responsibility for the decision to submit the article for publication. All authors approved the final version of the manuscript.

## Ethics Statement

All institutional and national guidelines for the care and use of laboratory animals were followed. Written consent from each patient was obtained.

## Consent

The authors have nothing to report.

## Conflicts of Interest

The authors declare no conflicts of interest.

## Supporting information


**Figure S1.** Knockdown of GLS1 does not significantly inhibit cancer cell proliferation. (A) WB validation of GLS1 knockdown using shRNA in MCF‐7 cells. (B) Cell proliferation of MCF‐7 cells with GLS1 knockdown. (C, D) Expression levels of GLS1 mRNA in breast cancer and colorectal cancer cell lines, based on data from the CCLE database (https://sites.broadinstitute.org/ccle).
**Figure S2**. Knockout of GLS1 significantly inhibited the proliferation of cancer cells, and supplementation with glutamate rescued cell proliferation. (A–C) Relative abundance of secreted ammonia, lactate, and cellular glucose of MCF‐7/Cas9 and MCF‐7/KOGLS1 cells. (D, E) Cell proliferation of LN229 cells with GLS1 knockout. (F) The colony formation assay was conducted on LN229 cells with GLS1 knockout, with or without 10 mM glutamate supplementation. (G) Cell proliferation of HCT116/Cas9 and HCT116/KOGLS1 cells supplemented with different concentrations of glutamate, α‐KG, and DMα‐KG (mM). All cultures were supplied with 10% dialyzed serum. Values are the means ± SEM of three independent experiments. *p < 0.05; ***p < 0.001; ****p < 0.0001 (Student’s *t*‐test).
**Figure S3**. Non‐essential amino acids that can be converted to glutamate can partially rescue the proliferation of GLS1 knockout cancer cells. (A, B) Cell proliferation of GLS1 knockout in MCF‐7 and HCT116 cells and control cells supplemented with branched‐chain amino acids and glutamate (mM). (C, D) Cell proliferation of GLS1 knockout in MCF‐7 and HCT116 cells supplemented with glutamate, or GABA (mM). (E) The impact of varying concentrations of glutamate, arginine, and proline (mM) on the cell proliferation of MCF‐7/Cas9 and MCF‐7/KOGLS1 cells. (F) The schematic diagram illustrates the interconversion process between glutamate and non‐essential amino acids. Values are the means ± SEM of three independent experiments. *p < 0.05; **p < 0.01; ***p < 0.001; ****p < 0.0001 (student’s *t*‐test).
**Figure S4**. Adding back of KGA and GAC can completely rescue the proliferation of GLS1 knockout cancer cells. (A, B) Influence of adding back KGA or GAC on the proliferation of Cas9, KOGLS1, KOGLS1/KGA, and KOGLS1/GAC cells under normoxia, hypoxia, and AntA. Values are the means ± SEM of three independent experiments.
**Figure S5**. Phospholipids cannot rescue the proliferation inhibition of cancer cells caused by knockout of GLS1. (A) The MSEA network of metabolites that decreased upon knockout of GLS1 was analysed with MetaboAnalyst 5.0. (B, C) Proliferation of Cas9 and GLS1 knockout cells cultured for 3 days in the presence of glutamate (mM), DMαKG (mM), or Lipid Mixture (100×). (D–H) Proliferation of Cas9 and GLS1 knockout cells cultured for 3 days in the presence of PG (μM), glutamate(mM), NEAA (100×), or PC (μM). (I) Proliferation of Cas9 and GLS1 knockout cells cultured for 3 days in the presence of NEAA (100×), U (100 μM), or a mixture of AUCG (100 μM). All cultures were supplied with 10% dialyzed serum. Values are the means ± SEM of three independent experiments. *p < 0.05; **p < 0.01; ***p < 0.001; ****p < 0.0001 (Student’s *t*‐test).
**Figure S6**. Phospholipids cannot rescue the clonogenic potential ability of cancer cells caused by knockout of GLS1. (A, B) Clone formation of MCF‐7/KOGLS1/KGA + GAC cultured in the presence of PG or PC (μM). (C, D) Clone formation number of HCT116/KOGLS1 and HCT116/KOGLS1/KGA + GAC cultured in the presence of glutamate (10 mM), PG (μM), Lipid Mixture (100×), PC (μM), NAC (0.5 mM) or AUCG (100 μM). All cultures were supplied with 10% dialyzed serum. Values are the means ± SEM of three independent experiments. *p < 0.05; **p < 0.01; ***p < 0.001 (Student’s *t*‐test).
**Figure S7**. Targeted metabolomics analysis of metabolic and gene regulatory changes upon GLS1 knockout. (A) Heatmap of metabolites clustering analysis of MCF‐7/KOGLS1 and MCF‐7/KOGLS1 + glutamate (10 mM) cultured for 8 h measured by LC–MS‐based targeted metabolomics. (B–H) Relative abundance of glutamate, arginine, proline, asparagine, ornithine, leucine, and serine in MCF‐7/KOGLS1/KGA + GAC, MCF‐7/KOGLS1 and MCF‐7/KOGLS1 cells with10 mM glutamate (Glu). (I) Fold change (log2) in metabolites after the addition of 10 mM glutamate to MCF‐7/KOGLS1 cells (MCF‐7/KOGLS1 vs. MCF‐7/KOGLS1 + Glu). All cultures were supplied with 10% dialyzed serum. Values are the means ± SEM of three independent experiments. *p < 0.05; **p < 0.01; ***p < 0.001; ****p < 0.0001 (Student’s *t*‐test).
**Figure S8**. Transcriptomics analysis of metabolic and gene regulatory changes upon GLS1 knockout. (A–C) Intracellular GSH, GSSG, and total GSH were measured in HCT116‐7/Cas9, HCT116/KOGLS1 cells with or without 10 mM glutamate. (D–I) Relative mRNA abundance of GLS1, GCLC, GSS, GPX4, MBOAT1 and MBOAT2 of Transcriptomics data. (J) KEGG enrichment was analysed for genes that were elevated in MCF‐7/KOGLS1 with 10 mM glutamate compared to MCF‐7/KOGLS1 cells. All cultures were supplied with 10% dialyzed serum. Values are the means ± SEM of three independent experiments. *p < 0.05; ****p < 0.0001 (Student’s *t*‐test).
**Figure S9**. GLS1 knockout leads to a notable decrease in the metabolic flux of glutamine towards GSH. (A, B) Relative abundance of GSH and GSSG in Cas9, KOGLS1, KOGLS1/KGA, KOGLS1/GAC and KOGLS1/KGA + GAC cells. (C, D) Relative abundance of glutamine and glutamate in Cas9, KOGLS1 and KOGLS1/KGA + GAC cells cultured for 8 h. (E, F) Mass isotopomer analysis of glutamine and γ‐glutamylcysteine in Cas9, KOGLS1, and KOGLS1/KGA + GAC cultured with the medium containing 1 mM of ^13^C_5_‐glutamine for 8 h. All cultures were supplied with 10% dialyzed serum. Values are the means ± SEM of three independent experiments. *p < 0.05; **p < 0.01; ***p < 0.001; ****p < 0.0001 (compared with KOGLS1) (Student’s *t*‐test).
**Figure S10**. GSH rescues proliferation depression of cancer cells owing to GLS1 knockout. (A) Clone formation of MCF‐7/KOGLS1 after the addition of GSH‐MEE (mM) to a conditioned medium. (B, C) Relative abundance of glutamate in media of Cas9, KOGLS1, KOGLS1/KGA, KOGLS1/GAC and KOGLS1/KGA + GAC cells. All cultures were supplied with 10% dialyzed serum. Values are the means ± SEM of three independent experiments. **p < 0.01; ***p < 0.001; ****p < 0.0001 (Student’s *t*‐test).
**Figure S11**. Knockout of GLS1 leads to impaired antioxidant capacity. (A) Detection of intracellular ROS levels in Cas9 and KOGLS1 cells. One hour prior to detection, 50 μM H_2_O_2_ was added to another group of Cas9 cells as a positive control. (B) Relative cell death after the addition of 50 μM H_2_O_2_ to condition media for 4 h. (C, D) Proliferation of MCF‐7/KOGLS1 cells with down‐regulated GCLC was detected after the addition of 10 mM glutamate to the conditioned medium. (E) Relative abundance of MDA in HCT116/Cas9, HCT116/KOGLS1, HCT116/KOGLS1/KGA, HCT116/KOGLS1/GAC and HCT116/KOGLS1/KGA + GAC cells. (F) Relative cell death after the addition of 5 μM erastin to condition media for 24 h. All cultures were supplied with 10% dialyzed serum. Values are the means ± SEM of three independent experiments. **p < 0.01; ***p < 0.001; ****p < 0.0001 (Student’s *t*‐test).
**Figure S12**. Knockout of GLS1 leads GPX4 downregulation but GPX1 upregulation. (A) Protein expressions were detected in HCT116/Cas9, HCT116/KOGLS1, HCT116/KOGLS1/KGA, HCT116/KOGLS1/GAC and HCT116/KOGLS1/KGA + GAC cells by Western blot. (B) Relative quantification of protein expression in the indicated cell lines (p‐AMPKα compared to total AMPKα, and other proteins compared to β‐actin). (C) Transcriptomic analysis and Gene Set Enrichment Analysis (GSEA) were conducted to identify differentially enriched pathways between KOGLS1(B) and KGA + GAC(A) cell lines. (D) Western blot analysis of AMPK and p‐AMPK expression in KOGLS1/KGA + GAC and KOGLS1 cells under different concentrations of BPTES. (E) Western blot analysis of GPX4 and GPX1 expression in MCF‐7/KOGLS1 and MCF‐7/KOGLS1/KGA + GAC cells under H_2_O_2_ stimulation at different concentrations. (F) Relative quantification of protein expression in the indicated cell lines (compared to β‐actin). The two groups of each cell line with H_2_O_2_ were compared to the group without H_2_O_2_, respectively. (G) 10 mg/mL CHX was added, with protein samples collected at different time points to examine GPX4 protein stability via Western blot. All cultures were supplied with 10% dialyzed serum. Values are the means ± SEM of three independent experiments. **p < 0.01; ***p < 0.001; ****p < 0.0001 (Student’s *t*‐test).
**Figure S13**. GLS1, GPX4 and GPX1 inhibitors synergistically suppress cancer cell growth. (A) GLS1 enzymatic activity assay was performed using a GLS1 activity detection kit in HCT116/Cas9, HCT116/KOGLS1, HCT116/KOGLS1/KGA, HCT116/KOGLS1/GAC, and HCT116/KOGLS1/KGA + GAC cells, following 12 h of culture in conditioned medium. (B) GLS1 enzymatic activity assay was conducted in MCF‐7/KGA + GAC cells after 12 h of treatment with varying concentrations of BPTES. (C–H) IC50 values of BPTES, ML‐210, and ED‐71 as single agents after 72 h of treatment. All cultures were supplied with 10% dialyzed serum. Values are the means ± SEM of three independent experiments. *p < 0.05; **p < 0.01; ***p < 0.001; ****p < 0.0001 (Student’s *t*‐test).
**Figure S14**. GLS1 and GPX4 are correlated with poor prognosis of cancer patients. (A, B) The survival analysis was performed on Kaplan–Meier plotter database.

**Table S1.** The metabolic flux of 13C5‐glutamine towards GSH in MCF‐7 cells (percentage).

**Table S2.** The metabolic flux of 13C5‐glutamine towards GSH in MCF‐7 cells (peak area).

**Table S3.** The metabolic flux of 13C5‐glutamine towards GSH in HCT116 cells (percentage).

**Table S4.** The metabolic flux of 13C5‐glutamine towards GSH in HCT116 cells (peak area).

## Data Availability

The data that support the findings of this study are available from the corresponding author upon reasonable request.
