# Supplementary material for: Glutaminase‐1 Mediated Glutaminolysis to Glutathione Synthesis Maintains Redox Homeostasis and Modulates Ferroptosis Sensitivity in Cancer Cells
Source: Cell Prolif. 2025 Apr 21;58(11):e70036. doi: 10.1111/cpr.70036 (PMC12584870; doi:10.1111/cpr.70036)

Figure S1

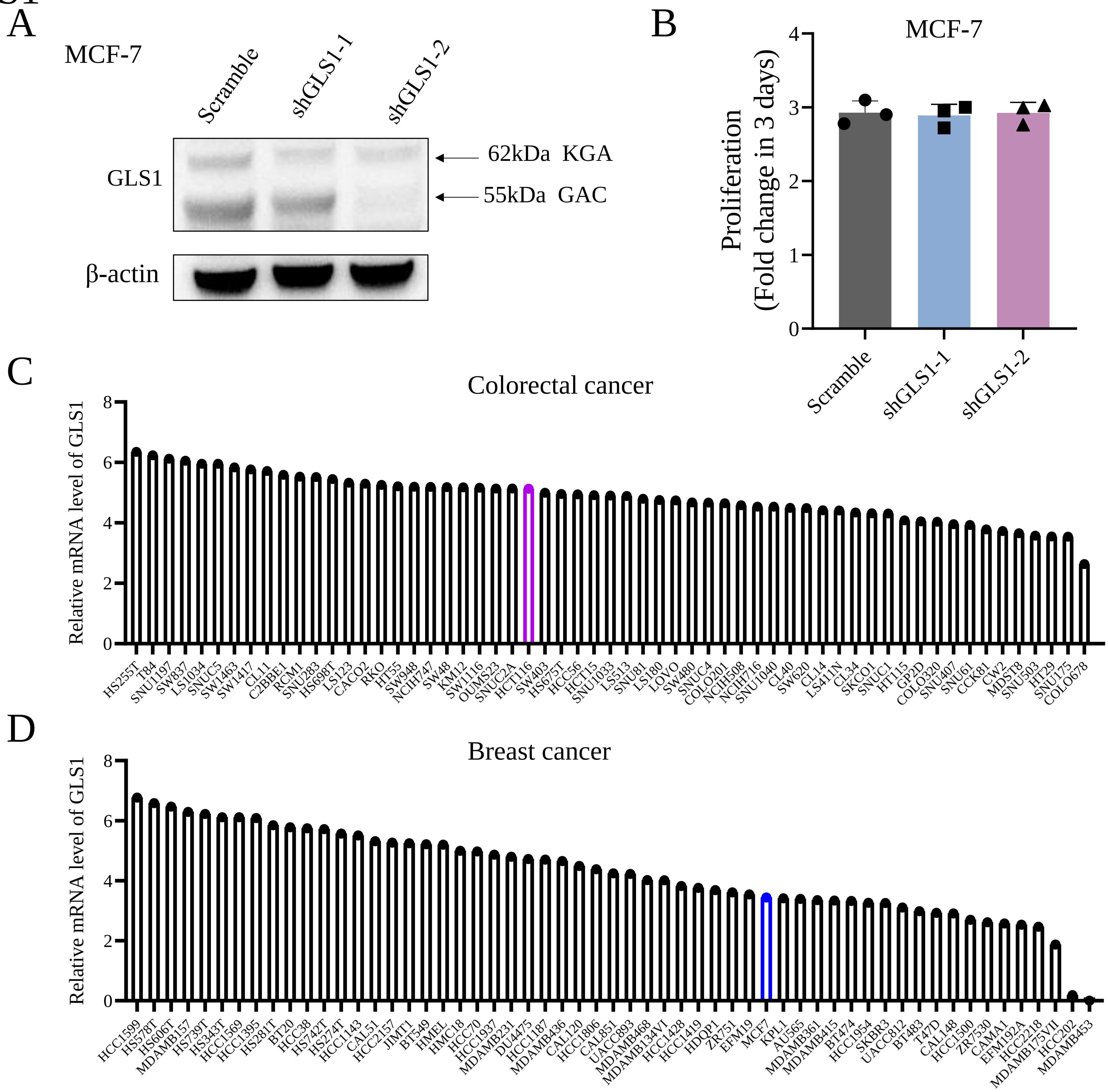
A
MCF-7
Scramble
shGLS1-1
shGLS1-2
GLS1
62kDa KGA
55kDa GAC
β-actin
B
MCF-7
Proliferation
(Fold change in 3 days)
0
1
2
3
4
Scramble
shGLS1-1
shGLS1-2
C
Colorectal cancer
Relative mRNA level of GLS1
0
2
4
6
8
HS255T
T84
SNU1197
SW837
LS1034
SNUC5
SW1463
SW1417
CL11
C2BBE1
RCM1
SNU283
HS698T
LS123
CACO2
RKO
HT55
SW948
NCIH747
SW48
KM12
SW1116
OUMS23
SNUC2A
HCT116
SW403
HS675T
HCC56
HCT15
SNU1033
LS513
SNU81
LS180
LOVO
SW480
SNUC4
COLO201
NCIH508
NCIH716
SNU1040
CL40
SW620
CL14
LS411N
CL34
SKCO1
SNUC1
HT115
GP2D
COLO320
SNU407
SNU61
CCK81
CW2
MDST8
SNU503
HT29
SNU175
COLO678
D
Breast cancer
Relative mRNA level of GLS1
0
2
4
6
8
HCC1599
HS578T
HS606T
MDAMB157
HS739T
HS343T
HCC1569
HCC1395
HS281T
BT20
HCC38
HS742T
HS274T
HCC1143
CAL51
HCC2157
JIMT1
BT549
HMEL
HMC18
HCC70
HCC1937
MDAMB231
DU4475
HCC1187
MDAMB436
CAL120
HCC1806
CAL851
UACC893
MDAMB468
MDAMB134VI
HCC1428
HCC1419
HDQP1
ZR751
EFM19
MCF7
KPL1
AU565
MDAMB361
MDAMB415
BT474
HCC1954
SKBR3
UACC812
BT483
T47D
CAL148
HCC1500
ZR7530
CAMA1
EFM192A
HCC2218
MDAMB175VII
HCC202
MDAMB453

Figure S2

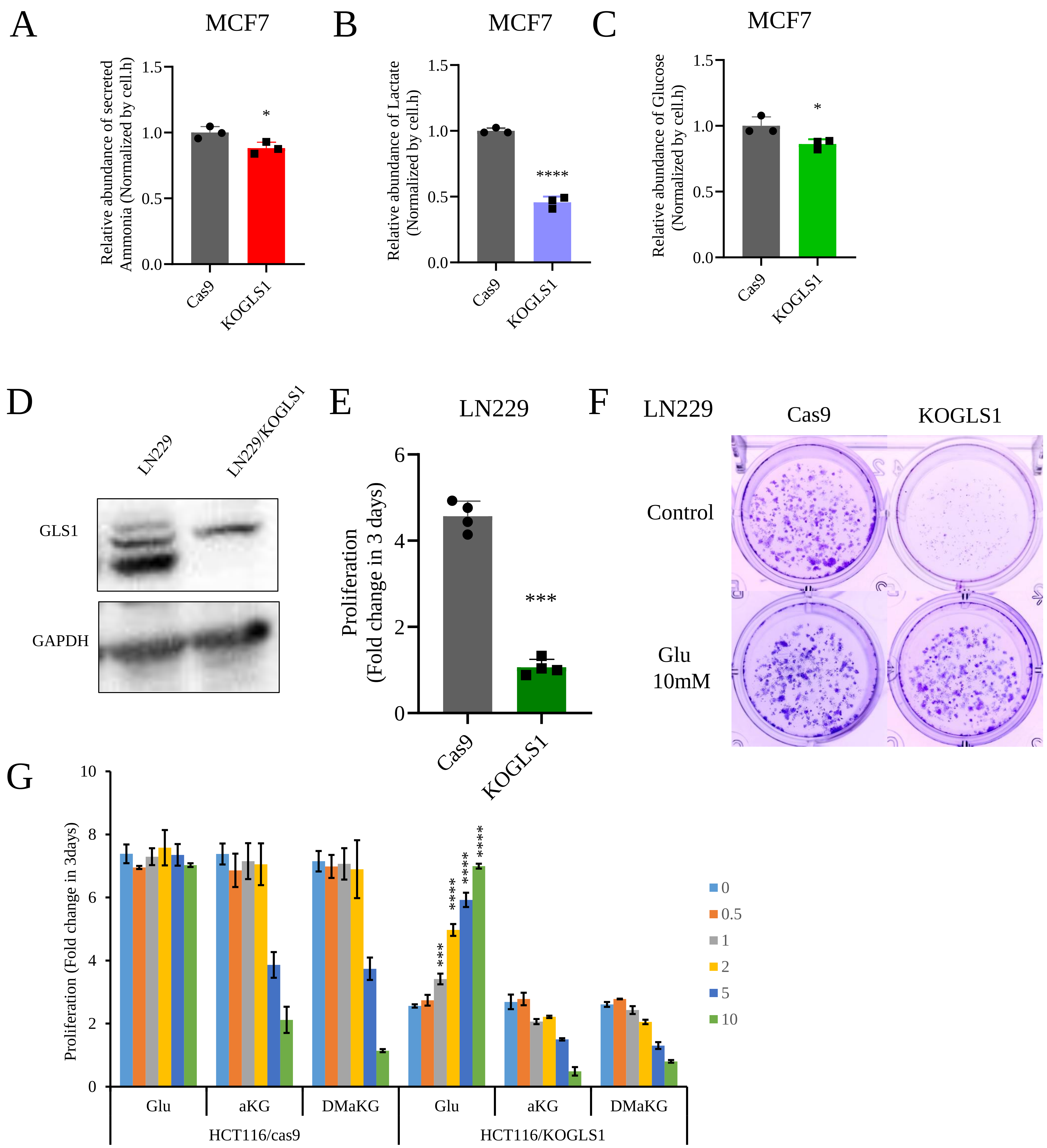

A
MCF7
Relative abundance of secreted Ammonia (Normalized by cell.h)
1.5
1.0
0.5
0.0
*
Cas9
KOGLS1
B
MCF7
Relative abundance of Lactate (Normalized by cell.h)
****
Cas9
KOGLS1
C
MCF7
Relative abundance of Glucose (Normalized by cell.h)
*
Cas9
KOGLS1
D
LN229
LN229/KOGLS1
GLS1
GAPDH
E
LN229
Proliferation (Fold change in 3 days)
6
4
2
0
***
Cas9
KOGLS1
F
LN229
Cas9
KOGLS1
Control
Glu 10mM
G
Proliferation (Fold change in 3days)
10
8
6
4
2
0
***
****
****
*****
Glu
aKG
DMaKG
Glu
aKG
DMaKG
HCT116/cas9
HCT116/KOGLS1
0
0.5
1
2
5
10

Figure S3

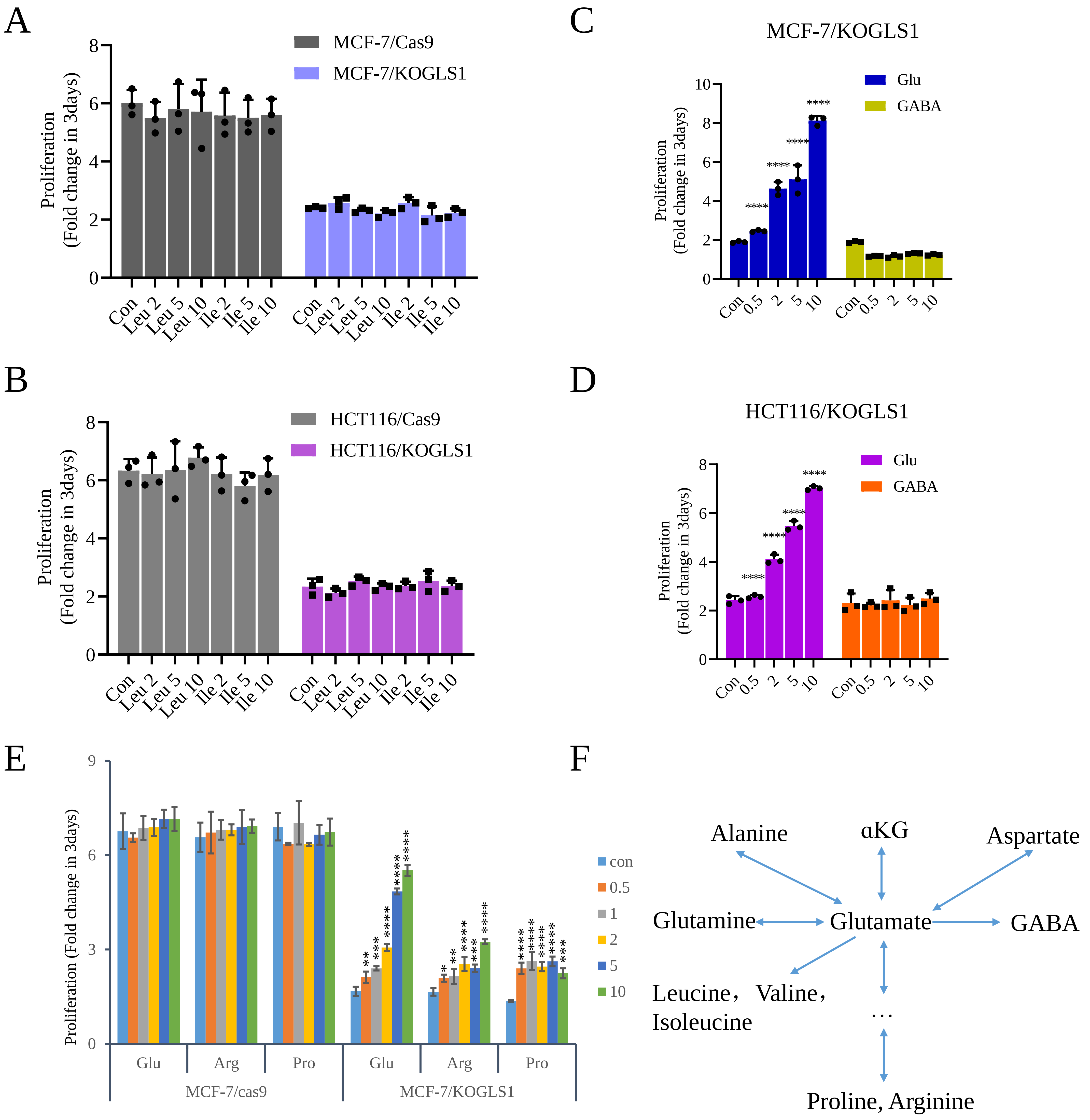
A
MCF-7/Cas9
MCF-7/KOGLS1
Proliferation (Fold change in 3days)
Con Leu 2 Leu 5 Leu 10 Ile 2 Ile 5 Ile 10
B
HCT116/Cas9
HCT116/KOGLS1
Proliferation (Fold change in 3days)
Con Leu 2 Leu 5 Leu 10 Ile 2 Ile 5 Ile 10
C
MCF-7/KOGLS1
Glu
GABA
Proliferation (Fold change in 3days)
Con 0.5 2 5 10
D
HCT116/KOGLS1
Glu
GABA
Proliferation (Fold change in 3days)
Con 0.5 2 5 10
E
Proliferation (Fold change in 3days)
con
0.5
1
2
5
10
Glu Arg Pro Glu Arg Pro
MCF-7/cas9
MCF-7/KOGLS1
F
Alanine
αKG
Aspartate
Glutamine
Glutamate
GABA
Leucine，Valine，
Isoleucine
…
Proline, Arginine

Figure S4

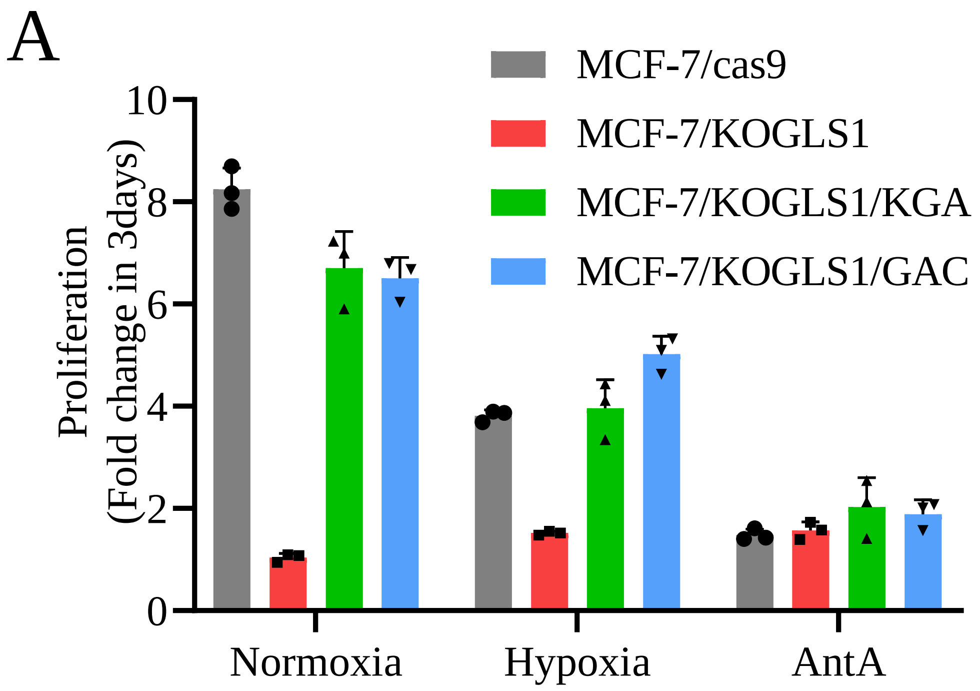
A
Proliferation
(Fold change in 3days)
10
8
6
4
2
0
Normoxia
Hypoxia
AntA
MCF-7/cas9
MCF-7/KOGLS1
MCF-7/KOGLS1/KGA
MCF-7/KOGLS1/GAC

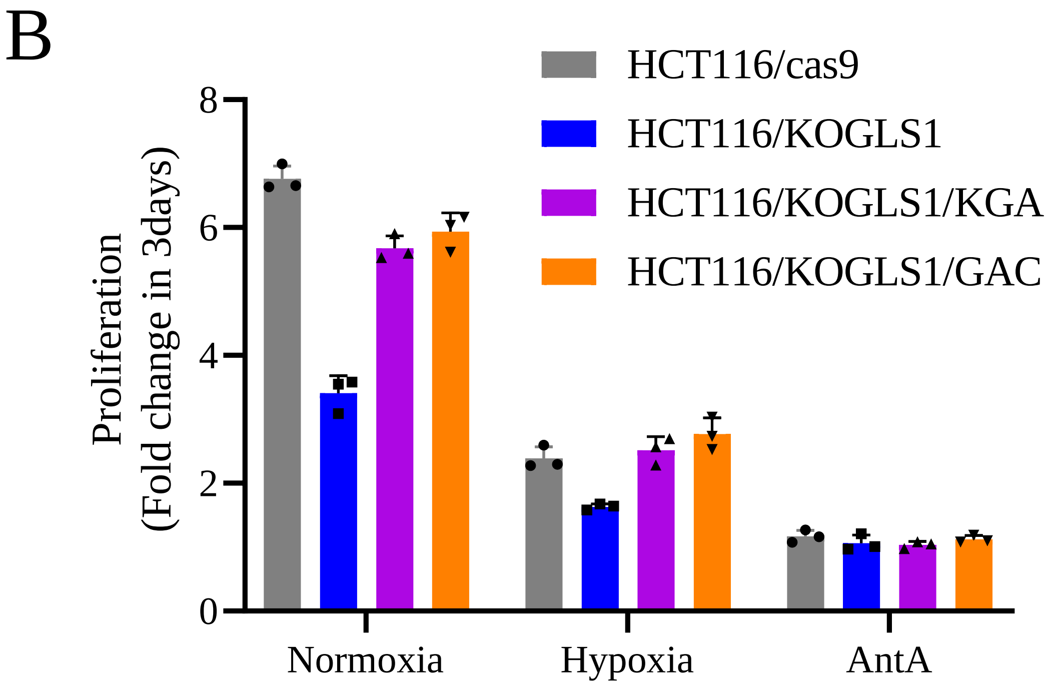
B
Proliferation
(Fold change in 3days)
8
6
4
2
0
Normoxia
Hypoxia
AntA
HCT116/cas9
HCT116/KOGLS1
HCT116/KOGLS1/KGA
HCT116/KOGLS1/GAC

Figure S5

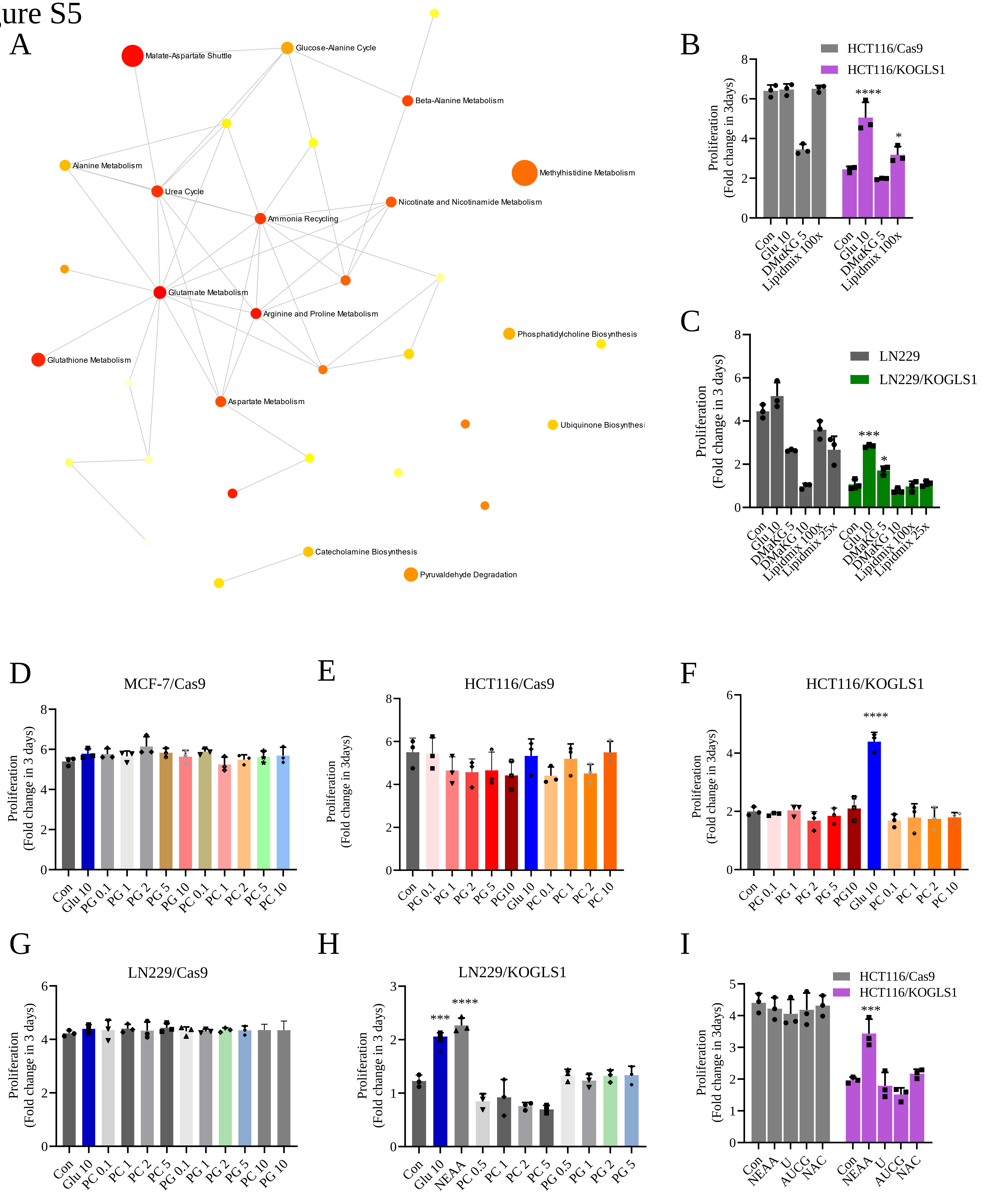
A
Malate-Aspartate Shuttle
Glucose-Alanine Cycle
Beta-Alanine Metabolism
Alanine Metabolism
Urea Cycle
Methylhistidine Metabolism
Nicotinate and Nicotinamide Metabolism
Ammonia Recycling
Glutamate Metabolism
Arginine and Proline Metabolism
Phosphatidylcholine Biosynthesis
Glutathione Metabolism
Aspartate Metabolism
Ubiquinone Biosynthesi
Catecholamine Biosynthesis
Pyruvaldehyde Degradation
B
HCT116/Cas9
HCT116/KOGLS1
Proliferation
(Fold change in 3days)
Con
Glu 10
DMαKG 5
Lipidmix 100x
C
LN229
LN229/KOGLS1
Proliferation
(Fold change in 3 days)
DMαKG 10
Lipidmix 25x
D
MCF-7/Cas9
PG 0.1
PG 1
PG 2
PG 5
PG 10
PC 0.1
PC 1
PC 2
PC 5
PC 10
E
HCT116/Cas9
PG10
F
HCT116/KOGLS1
G
LN229/Cas9
H
LN229/KOGLS1
NEAA
PC 0.5
PG 0.5
I
HCT116/Cas9
HCT116/KOGLS1
U
AUCG
NAC

Figure S6

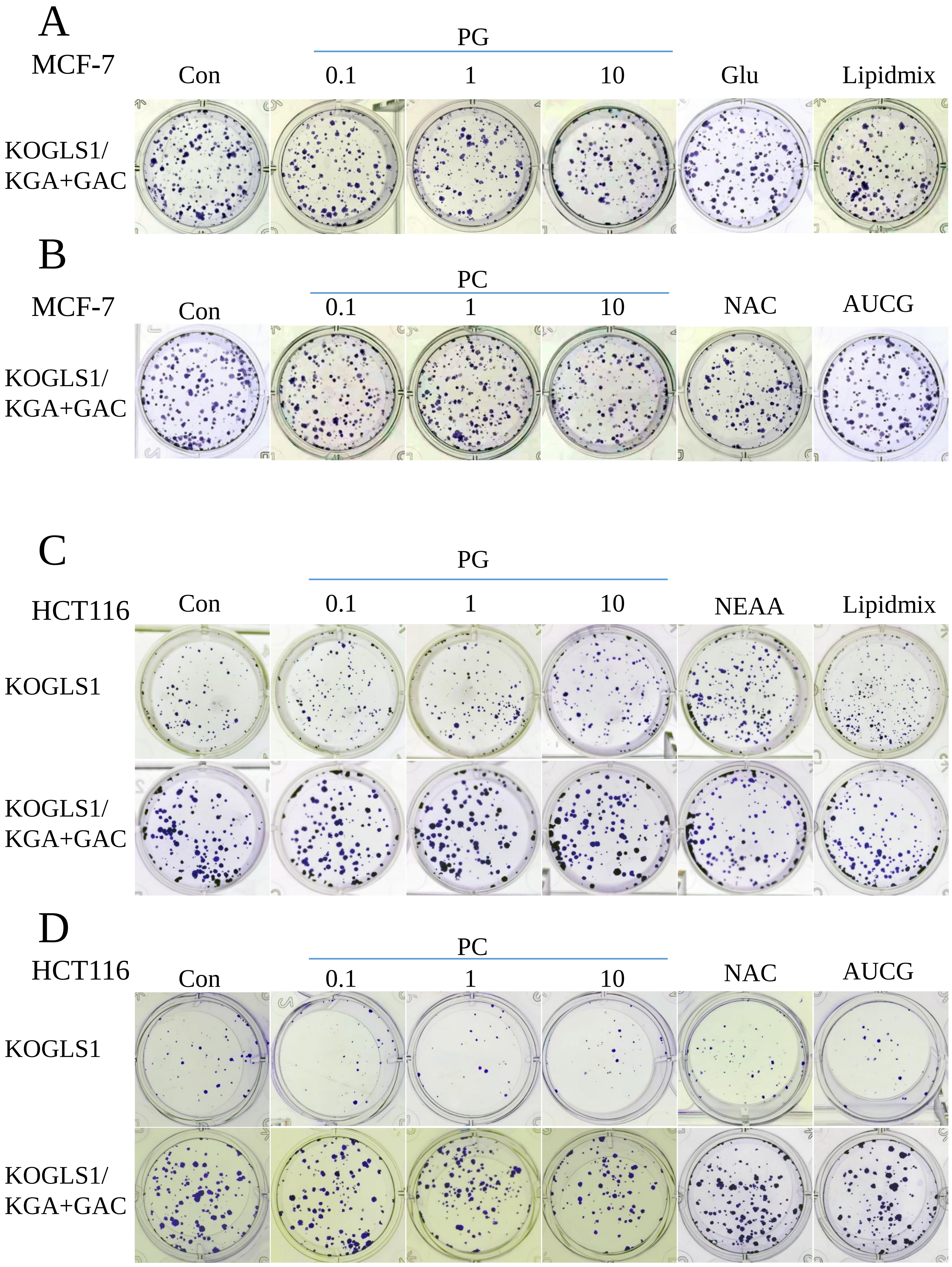
A
MCF-7
PG
Con
0.1
1
10
Glu
Lipidmix
KOGLS1/
KGA+GAC
B
MCF-7
PC
Con
0.1
1
10
NAC
AUCG
KOGLS1/
KGA+GAC
C
PG
HCT116
Con
0.1
1
10
NEAA
Lipidmix
KOGLS1
KOGLS1/
KGA+GAC
D
PC
HCT116
Con
0.1
1
10
NAC
AUCG
KOGLS1
KOGLS1/
KGA+GAC

Figure S7

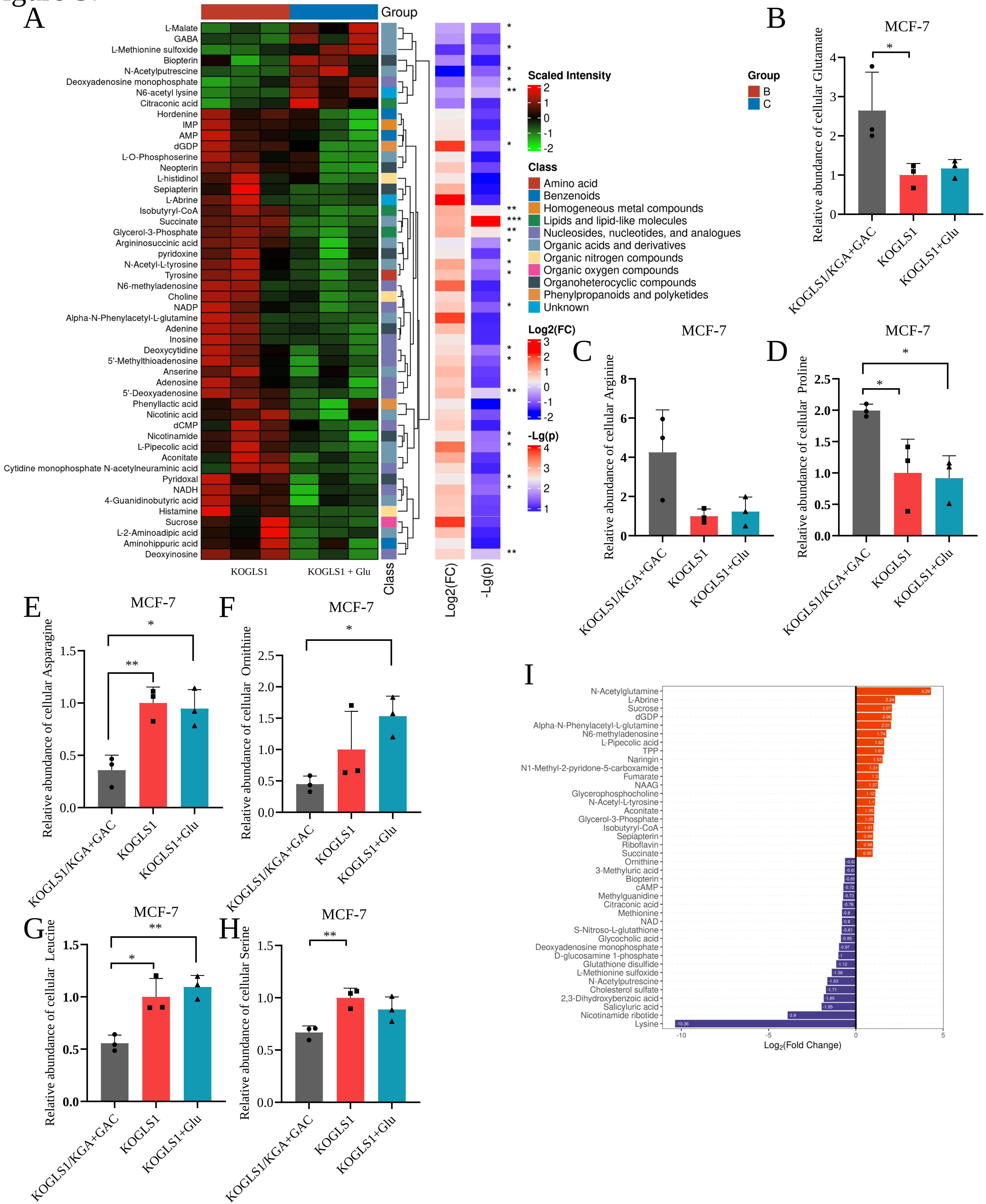

A
Group
L-Malate
GABA
L-Methionine sulfoxide
Biopterin
N-Acetylputrescine
Deoxyadenosine monophosphate
N6-acetyl lysine
Citraconic acid
Hordenine
IMP
AMP
dGDP
L-O-Phosphoserine
Neopterin
L-histidinol
Sepiapterin
L-Abrine
Isobutyryl-CoA
Succinate
Glycerol-3-Phosphate
Argininosuccinic acid
pyridoxine
N-Acetyl-L-tyrosine
Tyrosine
N6-methyladenosine
Choline
NADP
Alpha-N-Phenylacetyl-L-glutamine
Adenine
Inosine
Deoxycytidine
5'-Methylthioadenosine
Anserine
Adenosine
5'-Deoxyadenosine
Phenyllactic acid
Nicotinic acid
dCMP
Nicotinamide
L-Pipecolic acid
Aconitate
Cytidine monophosphate N-acetylneuraminic acid
Pyridoxal
NADH
4-Guanidinobutyric acid
Histamine
Sucrose
L-2-Aminoadipic acid
Aminohippuric acid
Deoxyinosine
KOGLS1
KOGLS1 + Glu
Class
Log2(FC)
-Lg(p)
Scaled Intensity
2
1
0
-1
-2
Group
B
C
Class
Amino acid
Benzenoids
Homogeneous metal compounds
Lipids and lipid-like molecules
Nucleosides, nucleotides, and analogues
Organic acids and derivatives
Organic nitrogen compounds
Organic oxygen compounds
Organoheterocyclic compounds
Phenylpropanoids and polyketides
Unknown
Log2(FC)
3
2
1
0
-1
-2
-Lg(p)
4
3
2
1
B
MCF-7
Relative abundance of cellular Glutamate
KOGLS1/KGA+GAC
KOGLS1
KOGLS1+Glu
C
MCF-7
Relative abundance of cellular Arginine
KOGLS1/KGA+GAC
KOGLS1
KOGLS1+Glu
D
MCF-7
Relative abundance of cellular Proline
KOGLS1/KGA+GAC
KOGLS1
KOGLS1+Glu
E
MCF-7
Relative abundance of cellular Asparagine
KOGLS1/KGA+GAC
KOGLS1
KOGLS1+Glu
F
MCF-7
Relative abundance of cellular Ornithine
KOGLS1/KGA+GAC
KOGLS1
KOGLS1+Glu
G
MCF-7
Relative abundance of cellular Leucine
KOGLS1/KGA+GAC
KOGLS1
KOGLS1+Glu
H
MCF-7
Relative abundance of cellular Serine
KOGLS1/KGA+GAC
KOGLS1
KOGLS1+Glu
I
N-Acetylglutamine
L-Abrine
Sucrose
dGDP
Alpha-N-Phenylacetyl-L-glutamine
N6-methyladenosine
L-Pipecolic acid
TPP
Naringin
N1-Methyl-2-pyridone-5-carboxamide
Fumarate
NAAG
Glycerophosphocholine
N-Acetyl-L-tyrosine
Aconitate
Glycerol-3-Phosphate
Isobutyryl-CoA
Sepiapterin
Riboflavin
Succinate
Ornithine
3-Methyluric acid
Biopterin
cAMP
Methylguanidine
Citraconic acid
Methionine
NAD
S-Nitroso-L-glutathione
Glycocholic acid
Deoxyadenosine monophosphate
D-glucosamine 1-phosphate
Glutathione disulfide
L-Methionine sulfoxide
N-Acetylputrescine
Cholesterol sulfate
2,3-Dihydroxybenzoic acid
Salicyluric acid
Nicotinamide ribotide
Lysine
-10
-5
0
5
Log2(Fold Change)

Figure S8

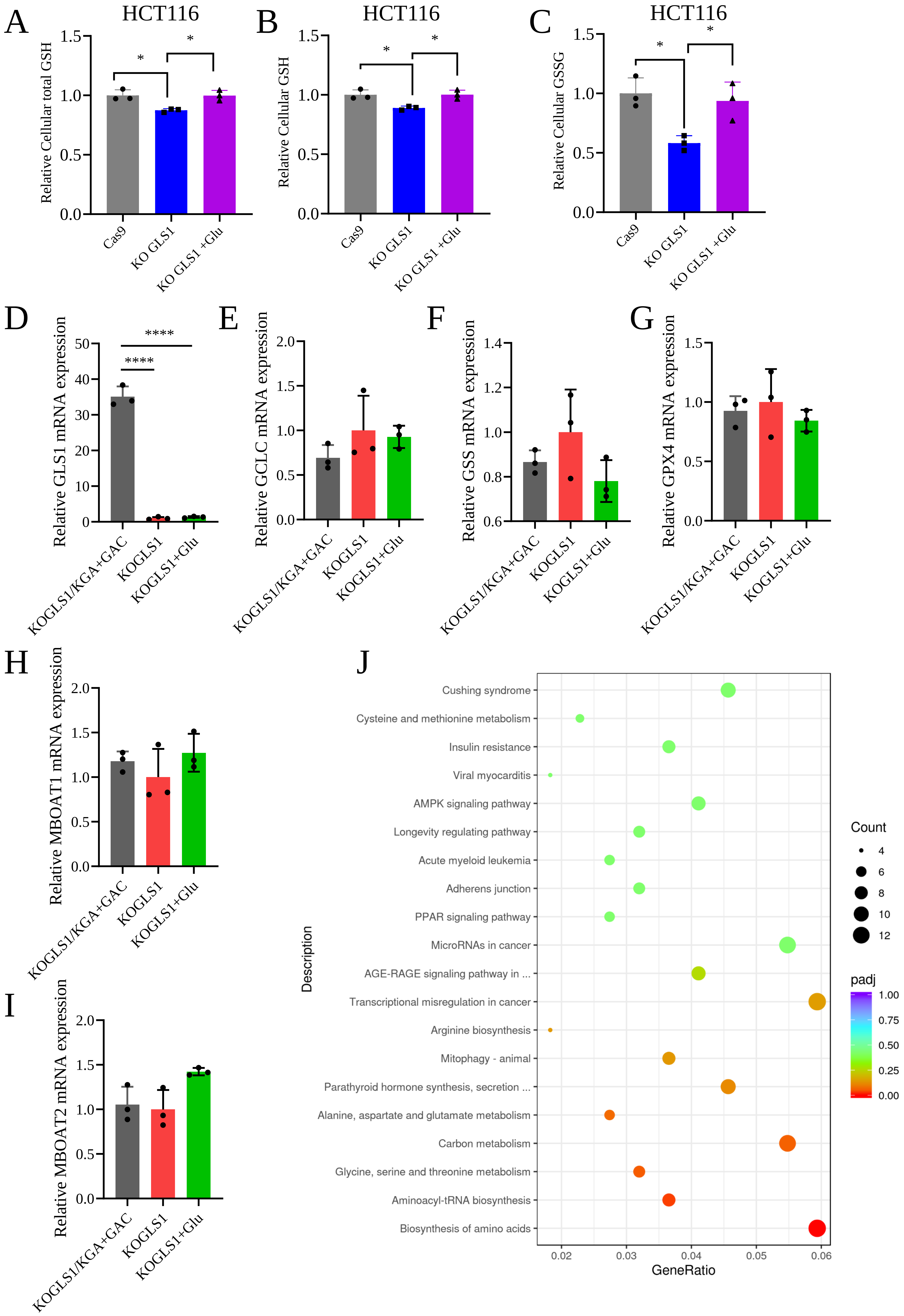

A
HCT116
Relative Cellular total GSH
Cas9
KO GLS1
KO GLS1 +Glu
B
HCT116
Relative Cellular GSH
C
HCT116
Relative Cellular GSSG
D
Relative GLS1 mRNA expression
KOGLS1/KGA+GAC
KOGLS1
KOGLS1+Glu
E
Relative GCLC mRNA expression
F
Relative GSS mRNA expression
G
Relative GPX4 mRNA expression
H
Relative MBOAT1 mRNA expression
I
Relative MBOAT2 mRNA expression
J
Cushing syndrome
Cysteine and methionine metabolism
Insulin resistance
Viral myocarditis
AMPK signaling pathway
Longevity regulating pathway
Acute myeloid leukemia
Adherens junction
PPAR signaling pathway
MicroRNAs in cancer
AGE-RAGE signaling pathway in ...
Transcriptional misregulation in cancer
Arginine biosynthesis
Mitophagy - animal
Parathyroid hormone synthesis, secretion ...
Alanine, aspartate and glutamate metabolism
Carbon metabolism
Glycine, serine and threonine metabolism
Aminoacyl-tRNA biosynthesis
Biosynthesis of amino acids
Description
GeneRatio
Count
padj

Figure S9

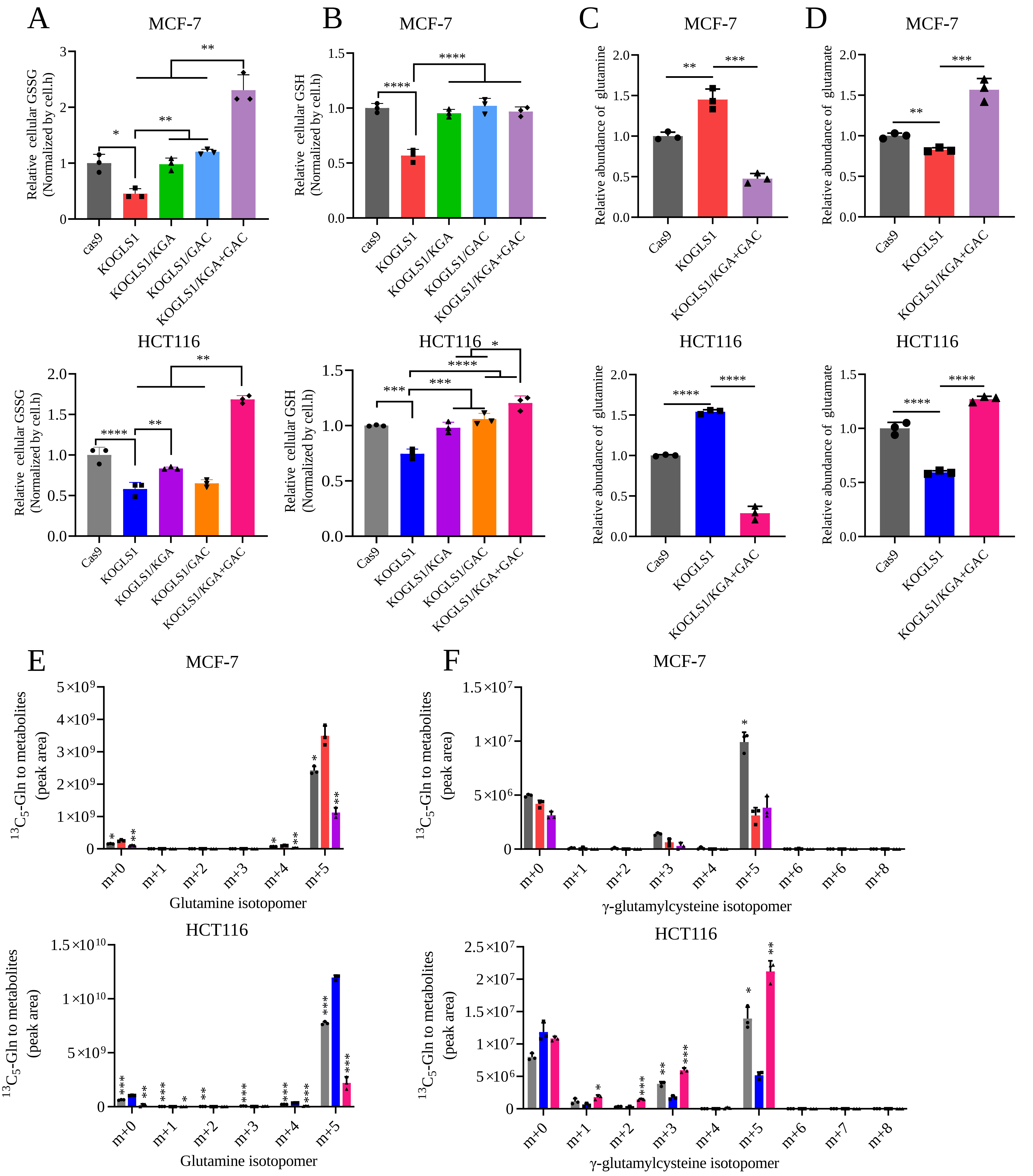

A
MCF-7
Relative cellular GSSG (Normalized by cell.h)
cas9
KOGLS1
KOGLS1/KGA
KOGLS1/GAC
KOGLS1/KGA+GAC
B
MCF-7
Relative cellular GSH (Normalized by cell.h)
C
MCF-7
Relative abundance of glutamine
Cas9
KOGLS1
KOGLS1/KGA+GAC
D
MCF-7
Relative abundance of glutamate
HCT116
E
MCF-7
$^{13}C_5$-Gln to metabolites (peak area)
Glutamine isotopomer
HCT116
F
MCF-7
γ-glutamylcysteine isotopomer
HCT116

Figure S10

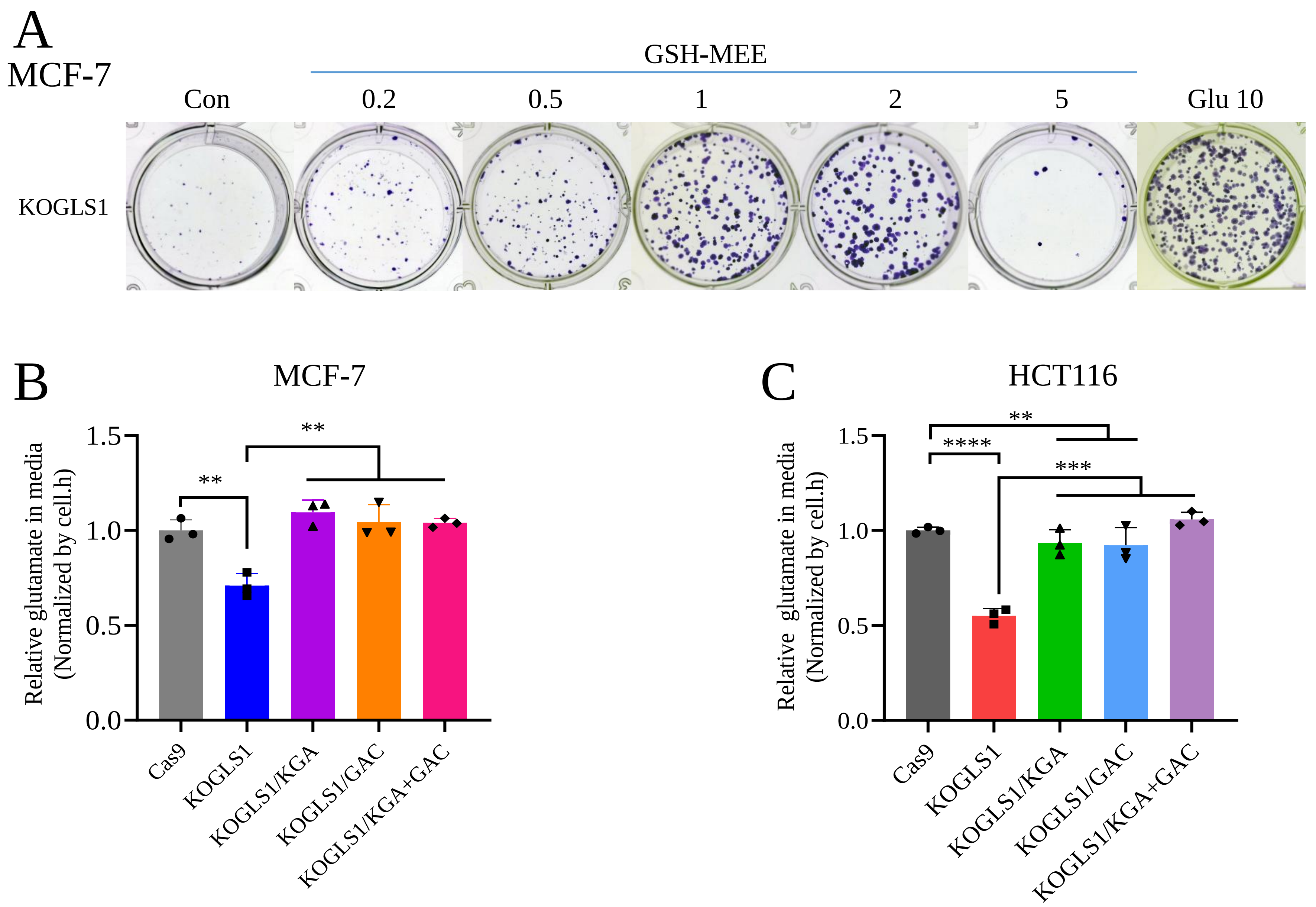
A
MCF-7
GSH-MEE
Con
0.2
0.5
1
2
5
Glu 10
KOGLS1
B
MCF-7
Relative glutamate in media (Normalized by cell.h)
1.5
1.0
0.5
0.0
**
**
Cas9
KOGLS1
KOGLS1/KGA
KOGLS1/GAC
KOGLS1/KGA+GAC
C
HCT116
Relative glutamate in media (Normalized by cell.h)
1.5
1.0
0.5
0.0
**
****
***
Cas9
KOGLS1
KOGLS1/KGA
KOGLS1/GAC
KOGLS1/KGA+GAC

Figure S11

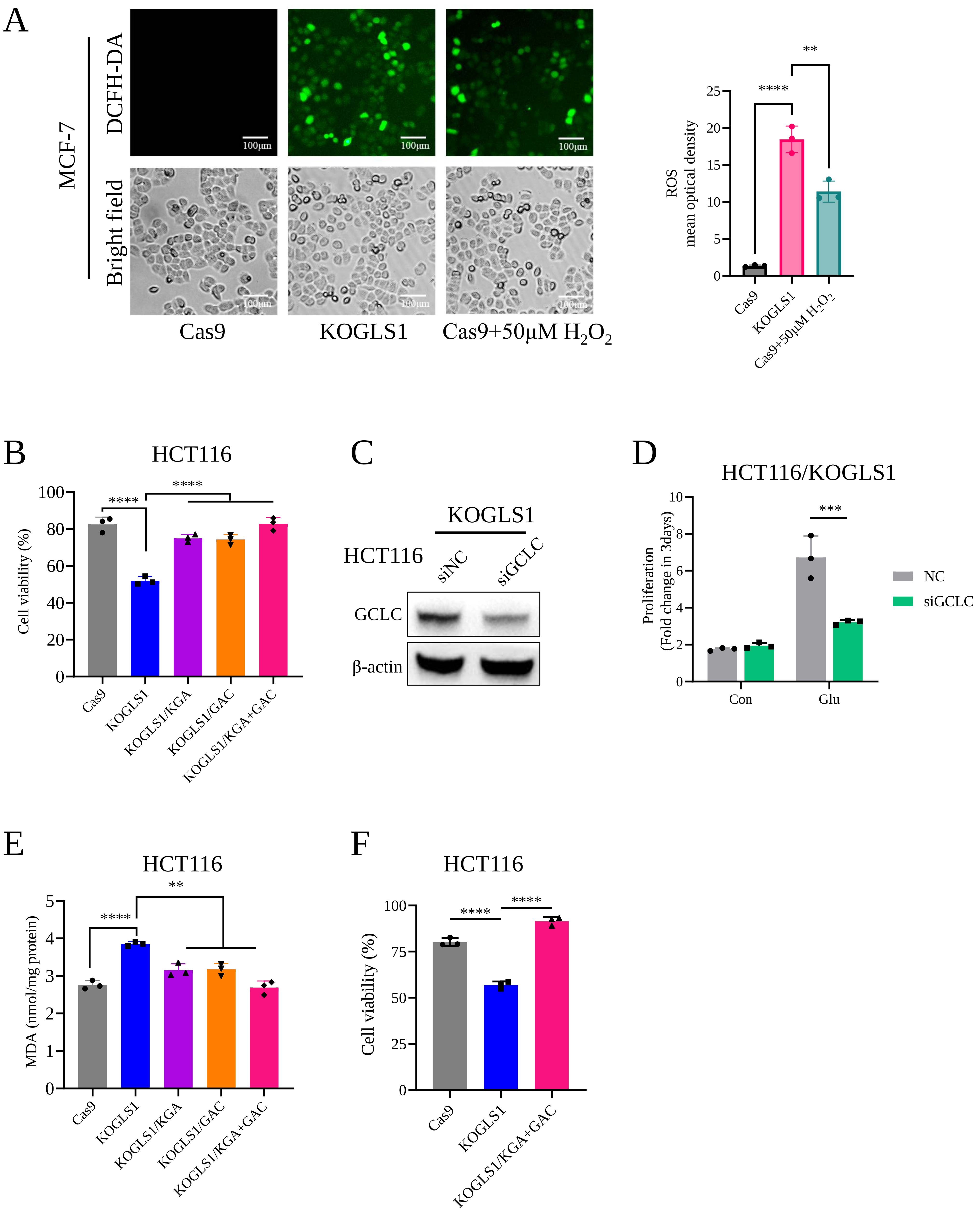
A
MCF-7
DCFH-DA
Bright field
100μm
Cas9
KOGLS1
Cas9+50μM H_2O_2
ROS
mean optical density
**
****
0
5
10
15
20
25
Cas9
KOGLS1
Cas9+50μM H_2O_2
B
HCT116
Cell viability (%)
****
****
0
20
40
60
80
100
Cas9
KOGLS1
KOGLS1/KGA
KOGLS1/GAC
KOGLS1/KGA+GAC
C
KOGLS1
HCT116
siNC
siGCLC
GCLC
β-actin
D
HCT116/KOGLS1
Proliferation
(Fold change in 3days)
***
0
2
4
6
8
10
Con
Glu
NC
siGCLC
E
HCT116
MDA (nmol/mg protein)
**
****
0
1
2
3
4
5
Cas9
KOGLS1
KOGLS1/KGA
KOGLS1/GAC
KOGLS1/KGA+GAC
F
HCT116
Cell viability (%)
****
****
0
25
50
75
100
Cas9
KOGLS1
KOGLS1/KGA+GAC

Figure S12

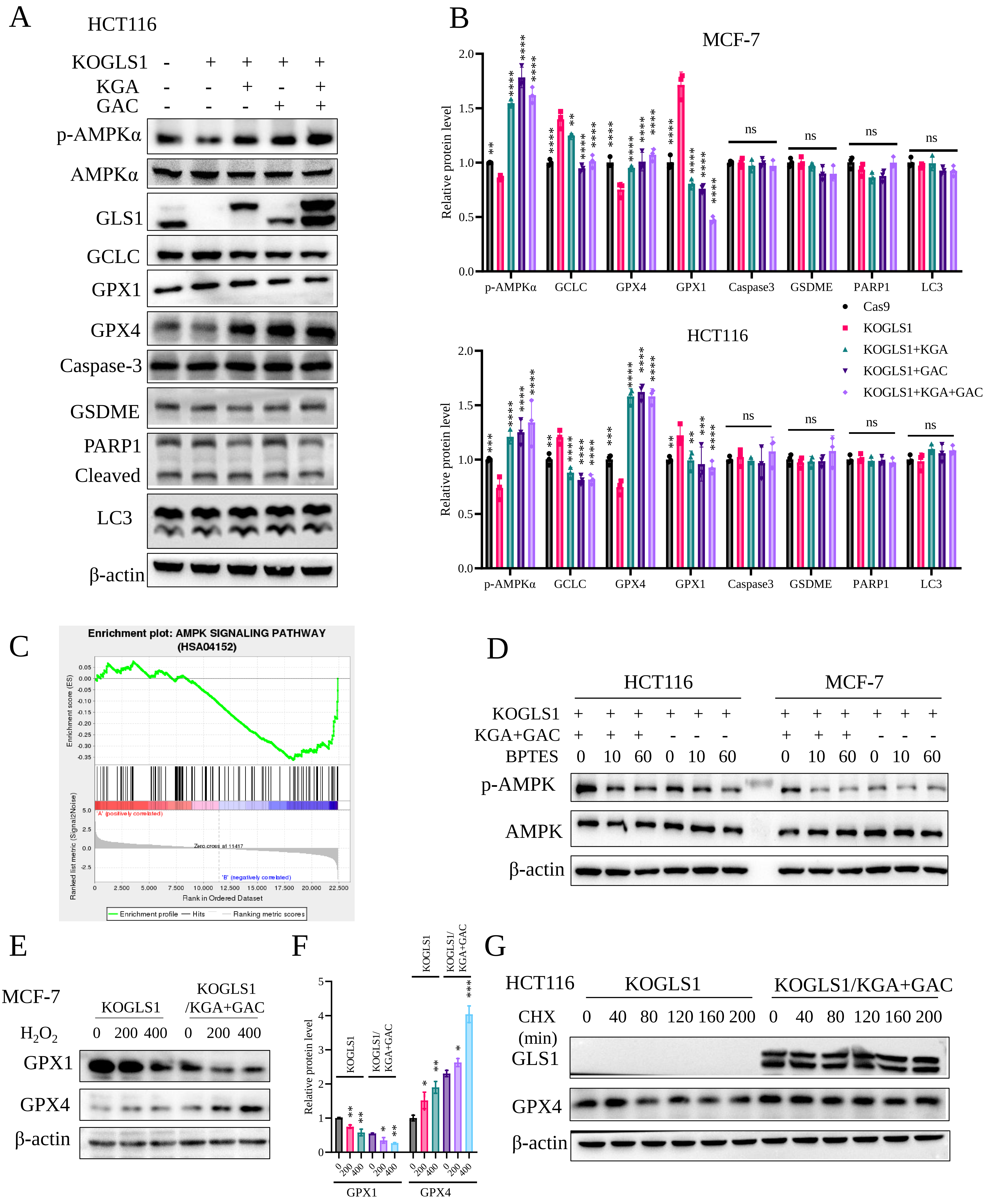

A
HCT116
KOGLS1 - + + + +
KGA - - + - +
GAC - - - + +
p-AMPKα
AMPKα
GLS1
GCLC
GPX1
GPX4
Caspase-3
GSDME
PARP1
Cleaved
LC3
β-actin
B
MCF-7
HCT116
Relative protein level
p-AMPKα
GCLC
GPX4
GPX1
Caspase3
GSDME
PARP1
LC3
ns
Cas9
KOGLS1
KOGLS1+KGA
KOGLS1+GAC
KOGLS1+KGA+GAC
C
Enrichment plot: AMPK SIGNALING PATHWAY (HSA04152)
Enrichment score (ES)
Ranked list metric (Signal2Noise)
'A' (positively correlated)
Zero cross at 11417
'B' (negatively correlated)
Rank in Ordered Dataset
Enrichment profile
Hits
Ranking metric scores
D
HCT116
MCF-7
KOGLS1 + + + + + + + + + + + +
KGA+GAC + + + - - - + + + - - -
BPTES 0 10 60 0 10 60 0 10 60 0 10 60
p-AMPK
AMPK
β-actin
E
MCF-7
KOGLS1
KOGLS1 /KGA+GAC
H2O2 0 200 400 0 200 400
GPX1
GPX4
β-actin
F
Relative protein level
KOGLS1
KOGLS1/ KGA+GAC
GPX1
GPX4
G
HCT116
KOGLS1
KOGLS1/KGA+GAC
CHX (min) 0 40 80 120 160 200 0 40 80 120 160 200
GLS1
GPX4
β-actin

Figure S13

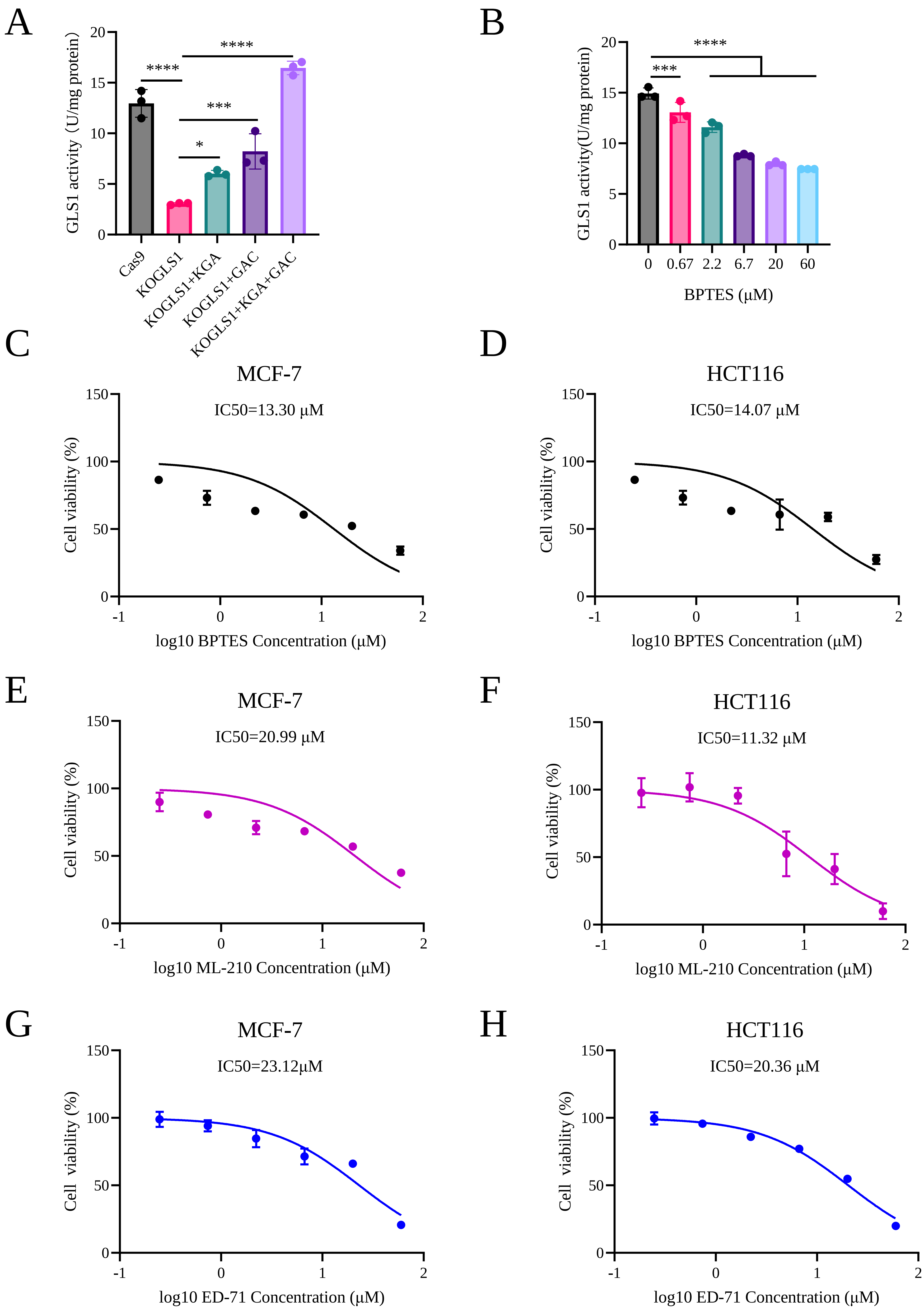

A
GLS1 activity (U/mg protein)
Cas9
KOGLS1
KOGLS1+KGA
KOGLS1+GAC
KOGLS1+KGA+GAC
B
GLS1 activity(U/mg protein)
BPTES (μM)
C
MCF-7
IC50=13.30 μM
Cell viability (%)
log10 BPTES Concentration (μM)
D
HCT116
IC50=14.07 μM
Cell viability (%)
log10 BPTES Concentration (μM)
E
MCF-7
IC50=20.99 μM
Cell viability (%)
log10 ML-210 Concentration (μM)
F
HCT116
IC50=11.32 μM
Cell viability (%)
log10 ML-210 Concentration (μM)
G
MCF-7
IC50=23.12μM
Cell viability (%)
log10 ED-71 Concentration (μM)
H
HCT116
IC50=20.36 μM
Cell viability (%)
log10 ED-71 Concentration (μM)

Figure S14

A

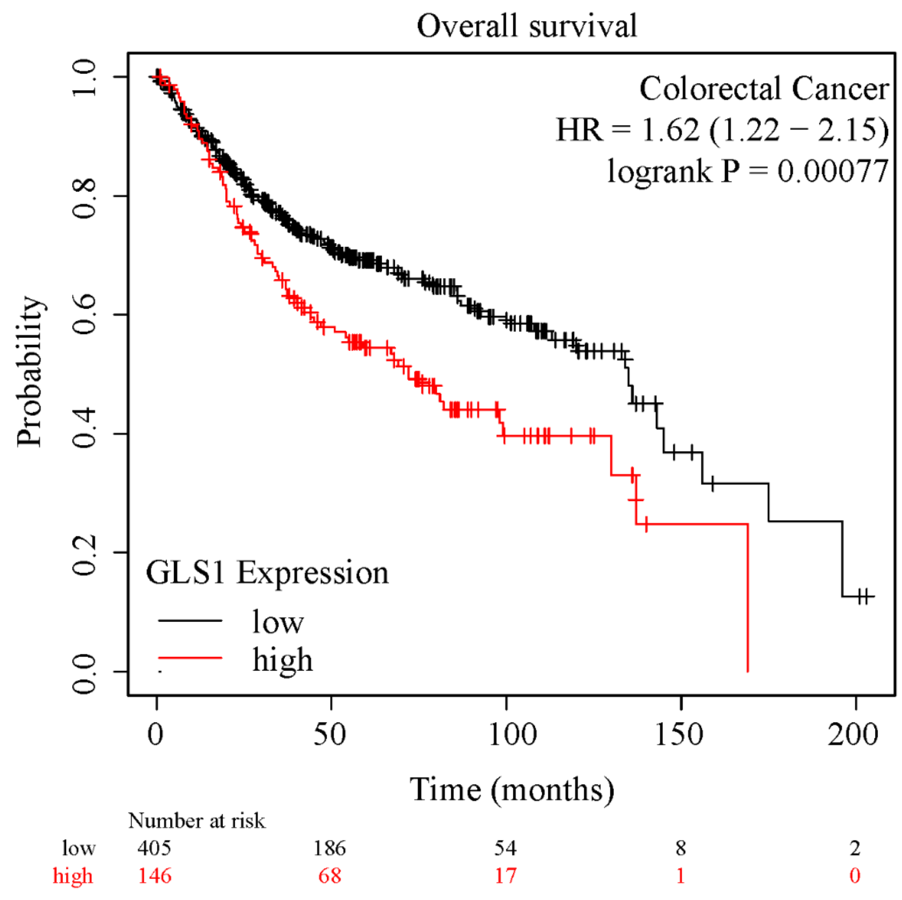

B

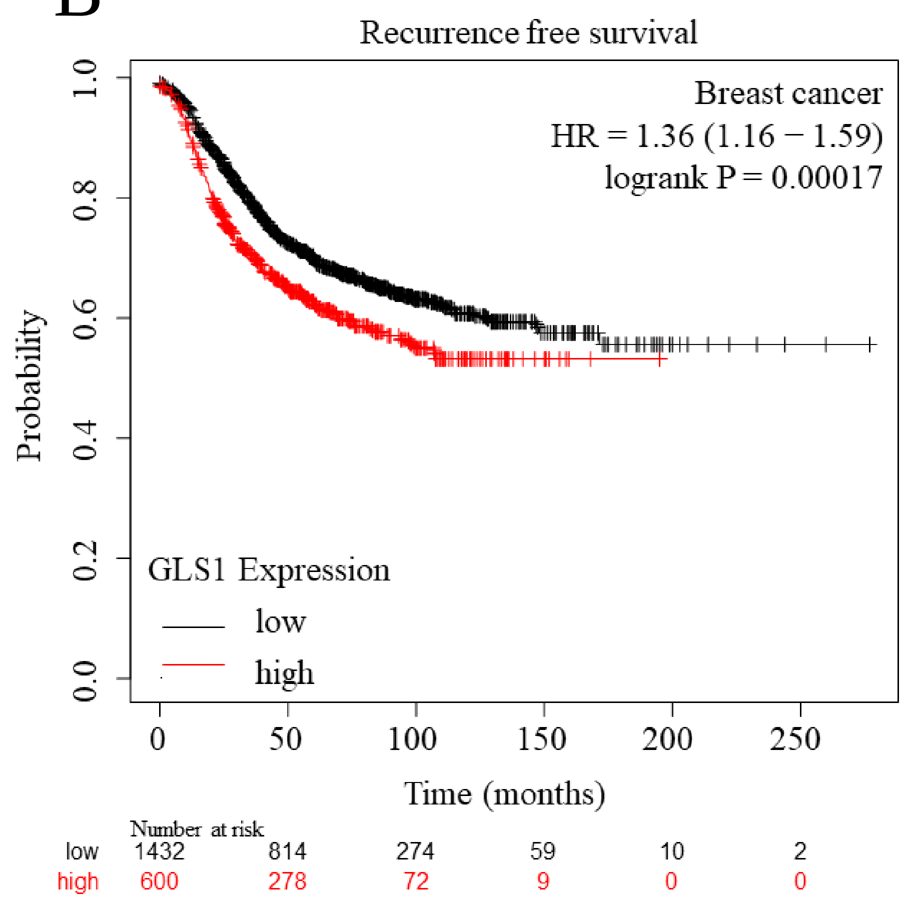

Supplement: Supplementary file 1 — Figure S1. Knockdown of GLS1 does not significantly inhibit cancer cell proliferation. (A) WB validation of GLS1 knockdown using shRNA in MCF‐7 cells. (B) Cell proliferation of MCF‐7 cells with GLS1 knockdown. (C, D) Expression levels of GLS1 mRNA in breast cancer and colorectal cancer cell lines, based on data from the CCLE database (https://sites.broadinstitute.org/ccle). Figure S2. Knockout of GLS1 significantly inhibited the proliferation of cancer cells, and supplementation with glutamate rescued cell proliferation. (A–C) Relative abundance of secreted ammonia, lactate, and cellular glucose of MCF‐7/Cas9 and MCF‐7/KOGLS1 cells. (D, E) Cell proliferation of LN229 cells with GLS1 knockout. (F) The colony formation assay was conducted on LN229 cells with GLS1 knockout, with or without 10 mM glutamate supplementation. (G) Cell proliferation of HCT116/Cas9 and HCT116/KOGLS1 cells supplemented with different concentrations of glutamate, α‐KG, and DMα‐KG (mM). All cultures were supplied with 10% dialyzed serum. Values are the means ± SEM of three independent experiments. *p < 0.05; ***p < 0.001; ****p < 0.0001 (Student’s t‐test). Figure S3. Non‐essential amino acids that can be converted to glutamate can partially rescue the proliferation of GLS1 knockout cancer cells. (A, B) Cell proliferation of GLS1 knockout in MCF‐7 and HCT116 cells and control cells supplemented with branched‐chain amino acids and glutamate (mM). (C, D) Cell proliferation of GLS1 knockout in MCF‐7 and HCT116 cells supplemented with glutamate, or GABA (mM). (E) The impact of varying concentrations of glutamate, arginine, and proline (mM) on the cell proliferation of MCF‐7/Cas9 and MCF‐7/KOGLS1 cells. (F) The schematic diagram illustrates the interconversion process between glutamate and non‐essential amino acids. Values are the means ± SEM of three independent experiments. *p < 0.05; **p < 0.01; ***p < 0.001; ****p < 0.0001 (student’s t‐test). Figure S4. Adding back of KGA and GAC can completely r [file CPR-58-e70036-s004.pdf]
